# Therapeutic Potential of Extracellular Vesicles (Exosomes) Derived From Platelet‐Rich Plasma: A Literature Review

**DOI:** 10.1111/jocd.16709

**Published:** 2024-12-01

**Authors:** Aditya K. Gupta, Tong Wang, Jeffrey A. Rapaport, Mesbah Talukder

**Affiliations:** ^1^ Division of Dermatology, Temerty Faculty of Medicine University of Toronto Toronto Ontario Canada; ^2^ Mediprobe Research Inc. London Ontario Canada; ^3^ Rapaport Hair Institute Englewood Cliffs New Jersey USA; ^4^ School of Pharmacy BRAC University Dhaka Bangladesh

**Keywords:** alopecia, cosmetic dermatology, wound healing

## Abstract

**Background:**

There are exciting advances in the field of exosomes research as a potential regenerative therapy. Platelet‐rich plasma represents an abundant source of exosomes.

**Aim:**

To present evidence on the use of platelet‐rich plasma‐derived exosomes in dermatology and discuss current technical limitations.

**Methods:**

A literature search was conducted using Embase (Ovid), Web of Science, and ClinicalTrials.gov in June 2024.

**Results:**

Platelet‐rich plasma‐derived exosomes contain a myriad of growth factors, genetic materials, and lipids that mediate paracrine signaling. Several preclinical studies have demonstrated their potential in wound healing. This includes faster wound contraction in diabetic animal models, as well as enhanced angiogenesis, increased cell proliferation and migration, and protection against hyperglycemic conditions in vitro. A case study reported a topical platelet‐derived exosome product tried successfully in a patient with persistent scalp wounds. In contrast, there is scarce information on the use of platelet‐rich plasma‐derived exosomes in hair growth and skin rejuvenation. Isolation techniques, activation methods, and methods of delivery have not been optimized, which warrants further research. Novel delivery methods, such as hydrogel‐based preparations, may enable the topical application of exosomes.

**Conclusion:**

Exosomes derived from platelet‐rich plasma have demonstrated therapeutic potential. More research is needed to standardize its use.

## 1 Introduction

Platelet‐rich plasma (PRP) is an autologous treatment that entails obtaining a blood sample through venipuncture followed by the separation of plasma enriched in platelets; subsequently, it is administered intradermally or subcutaneously [1]. This procedure has been popularized as a regenerative therapy due to its effects on hair growth [1], stimulation of collagen synthesis [2], wound healing, and anti‐inflammatory effects [3]. The therapeutic potential of PRP lies in the platelet production and secretion of growth factors, cytokines, chemokines, and extracellular vesicles (EVs) [4], which can be stimulated using various activation methods. Despite its popularity, there are significant heterogeneities in how PRP is processed and administered, which limits the comparability between studies [4]. To date, PRP has not been approved by the U.S. FDA; however, several PRP collection systems have received U.S. FDA 510 (k) clearance as medical devices.

**FIGURE 1:**
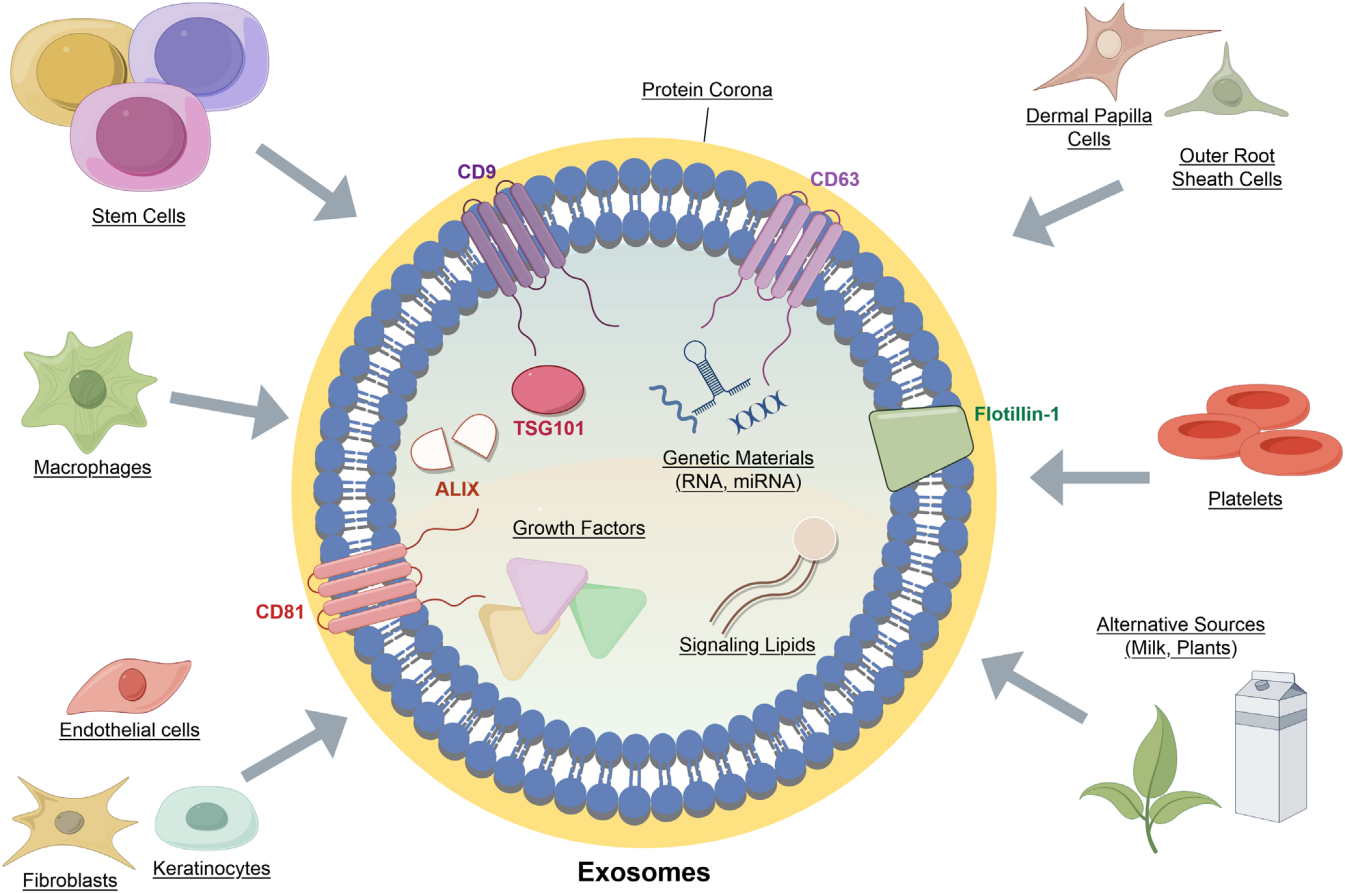
An overview of the biological characteristics of exosomes. Exosomes can be identified through their size (30–150 nm) and molecular markers (CD9, CD63, CD1, flotillin‐1, TSG101, Alix) [6]. Exosomes can be extracted from cells, non‐cells (platelets), and alternative sources (milk, plant extracts) [6, 7, 8]. Exosome cargos include growth factors, genetic materials, and lipids that function in wound‐healing, hair growth, collagen synthesis and regulating inflammation [6, 7, 8]; the type of cargo contained in exosomes is reflective of their origin. The protein corona—molecules that adhere around exosomes from its environment—also influences their characteristics and function [28]. Illustration created using Figdraw.

Exosomes are acellular, nonreplicative, nanosized EVs that mediate cell‐to‐cell communication through the delivery of cargos—such as growth factors, signaling lipids, and genetic materials—protected by their lipid bilayer (Figure 1). Despite a surge in research interest, its clinical translation is hampered due to technical limitations and a lack of standardized methodologies, as well as the diversity of exosome content reflective of its cellular origin [5]. Exosomes are secreted by all cell types and can be detected in a variety of bodily fluids [6, 7, 8]; in particular, allogeneic platelets have been discussed as a preferable source due to the abundance of exosomes, reduced immunogenicity, and ease of sample collection [9, 10], as opposed to the isolation of exosomes from stem cells where an additional ex vivo expansion step may be required to obtain sufficient quantities. Furthermore, standardized protocols and existing infrastructures for platelet isolation are available under the tenets of Good Manufacturing Practice [9], which help minimize safety concerns.

There is an increasing level of recognition of exosomes as a potential treatment for androgenetic alopecia [1, 8], skin aging [11], and wound healing [6, 7]. A review of all potential dermatology applications of EVs was published recently by Novis and colleagues [12]. Navigating the regulatory landscape of exosomes is a challenge due to its inherent complexity (e.g., difficulties in isolation, purification, and quality control; unclear mechanism of action), a wide range of potential clinical applications, and serious safety concerns regarding empirical use (explained further in Section 3.4) [13, 14]. At present, exosomes are not approved by the U.S. Food and Drug Administration (FDA) or the European Medicines Agency (EMA). The U.S. FDA has granted Investigational New Drug (IND) designations to selected exosome products [15]; however, use of such products in routine care and direct‐to‐consumer marketing is not allowed.

In this review, we aim to summarize available preclinical and clinical evidence on the therapeutic potential of *PRP‐ or platelet‐derived exosomes*. New perspectives on addressing the current technical challenges, such as combined isolation methods, the addition of platelet activators, and embedding exosomes in topical hydrogel preparations, are discussed.

## 2 Methods

An electronic literature search was conducted using EMBASE (Ovid) and Web of Science in June 2024. The search string included the following terms: “platelet‐rich plasma,” “thrombocyte rich plasma,” “dermatology,” “skin,” “hair,” “alopecia,” “face,” “burn,” “wound healing,” “exosome,” “microvesicle,” and “extracellular vesicle.” A search on ClinicalTrials.gov was conducted in June 2024 using the terms “platelet extracellular vesicles” and “platelet exosomes.” The references list of review articles was screened. No date or language restrictions were applied.

The inclusion criteria were full‐length articles reporting on the effects of *PRP‐derived exosomes* in hair loss, facial rejuvenation, burns, or wound healing. Studies that processed PRP into platelet lysate were considered. Due to the unstandardized nomenclature [16], a study was included if the terms “microvesicle” and “extracellular vesicle” were used with additional molecular characterizations. Articles without full‐text availability, preclinical studies that cannot be linked to the aforementioned conditions, reviews, and expert opinions were excluded. The following elements were recorded for data extraction: sample source, platelet concentration, exosome concentration, activation method, exosome diameter, surface markers and cargo, model system, and observed genotypic/phenotypic effects.

Since EV research has mostly remained in the preclinical stage, there are still significant heterogeneities in how EVs or exosomes are isolated and characterized across studies. At present, the International Society for Extracellular Vesicles (ISEV) does not mandate any particular experimental designs for EV research [16]. Quality of reporting was assessed using the EV‐Checklist developed by Poupardin et al. [17] in accordance with MISEV (Minimal Information for Studies of Extracellular Vesicles) set by ISEV. The following EV parameters were assessed: source (collection and storage), isolation, characterization, cargo, and function.

## 3 Results and Discussion

The literature search yielded 14 preclinical studies on the application of *PRP‐derived exosomes* in wound healing (*n* = 12), hair growth (*n* = 2), and skin rejuvenation (*n* = 1), which are summarized in Tables 1 and 2 [18, 19, 20, 21, 22, 23, 24, 25, 26, 27, 28, 29, 30, 31]. One case report was identified on the use of a *platelet‐derived exosomes* product in wound healing [32]. Platelet concentrations and additional characteristics of exosomes are presented as Supporting Information (Table S1).

**TABLE 1:** Investigations of PRP‐derived exosomes for wound healing.

Study	Sample source	Target	Phenotypic/Genotypic effects^a^	Activation	Content
Chen 2023 (1) [18]	Human PRP	Endothelial cells	Increase cell viability for migration Increase tube formation capacity	Thrombin and calcium gluconate (10:1)	Sphingosine‐1‐phosphate
		Wounded diabetic rat	Faster wound healing Improve re‐epithelialization; no change in collagen deposition Enhance angiogenesis Increase levels of FN1, p‐AKT, and VEGF‐A		
Rui 2024 [19]	Human PRP (apheresis)	Wounded diabetic mice	Faster wound healing Reduce number of infiltrating neutrophils forming neutrophil extracellular traps via inhibition of MMP‐8 Reduce number of apoptotic cells	Thrombin and calcium gluconate (10:1)	miRNA‐26b‐5p
Bakadia 2023 [24]	Rat PRP (cardiac blood)	Endothelial cells^b^ and Fibroblasts^b^	Increase cell viability and migration	Thrombin and calcium gluconate (1:1)	—
		Wounded diabetic rat^b^	Faster wound healing Enhance angiogenesis Formation of granulation tissues Improve re‐epithelialization and collagen deposition Increase cell proliferation Decrease in macrophages and neutrophil extracellular traps *Upregulate IGF‐1*, *TGF‐β1*, *EGF*, *VEGF* *Downregulate of MMP‐9*		
Cao 2023 [25]	Human PRP	Fibroblasts	Increase cell viability and migration Under a high glucose condition: rescue cell viability and migration, attenuate apoptosis Increase PDGF receptor Increase phosphorylation of JAK2 and STAT3 *Upregulate Bcl‐2* *Downregulate Bax*	Thrombin and CaCl_2_	PDGF‐BB VEGF b‐FGF
		Wounded diabetic rat^b^	Faster wound healing Improve re‐epithelialization, collagen deposition and arrangement Enhance angiogenesis		
Chen 2023 (2) [26]	Human PRP	Fibroblast cells	Increase cell viability Decrease apoptotic cells Inhibit pyroptosis and pro‐inflammatory cytokines *Upregulate MALAT1 and DNMT3A* *Downregulate miR‐374a‐5p*	Thrombin and CaCl_2_	—
Lovisolo 2020 [27]	Human PRP	Keratinocytes	Increase cell migration	CaCl_2_ and Calcimycin	—
Gnomes 2022 [28]	Human platelet lysate	Fibrospheres	Support 3D fibroblast spheroid formation	CaCl_2_	—
		Skin organoid model (keratinocytes, fibroblasts, epithelial cells)	Support 3D organoid formation Enhance angiogenesis		
		Fibroblast cells	Increase cell migration		
		T cells	Inhibit cell proliferation		
Guo 2017 [29]	Human PRP	Endothelial cells and Fibroblasts	Increase cell viability and migration Increase tube formation capacity Promote YAP dephosphorylation and upregulates CTGF Increase phosphorylation of AKT and Erk	None	PDGF‐BB TGF‐β b‐FGF VEGF
		Wounded diabetic rat^b^	Faster wound healing Enhance angiogenesis Improve re‐epithelialization, collagen deposition and arrangement		
Shu 2023 [30]	Human PRP	Endothelial cells^b^ and Fibroblast cells^b^	Under a high glucose condition: rescue cell proliferation, maintain autophagy and attenuate apoptosis	None	—
		Wounded diabetic rat^b^	Faster wound healing Improve re‐epithelialization, collagen deposition and arrangement Enhance angiogenesis Promote autophagy Inhibit apoptosis		
Yi 2023 [31]	Human PRP	Schwann cells	Increase proliferation and migration Increase section of growth/neurotrophic factors (NGF, VEGF, GDNF, BDNF) Upregulate pathways involved in peripheral nerve regeneration and repair	None	—
		Rat with sciatic nerve crush injury^c^	Improve toe mobility Improve SFI Recovery of nerve conduction Nerve regeneration and reversal and muscle atrophy		
Xu 2018 [20]	Human PRP	Wounded diabetic rat	Faster wound healing Reduce number of skin ulcers Increase epidermal thickness Improve collagen synthesis and deposition Enhance angiogenesis	—	—

Abbreviations: AKT, protein kinase; B; Bax; Bcl‐2, B‐cell lymphoma type 2; Bcl‐2‐associated X protein; BDNF, brain‐derived neurotrophic factor; b‐FGF, basic fibroblast growth factor; CTGF, connective tissue growth factor; DNMT3A, DNA methyltransferase 3 alpha; EGF, endothelial growth factor; Erk, extracellular signal‐regulated kinase; FN1, fibronectin 1; GDNF, glial cell‐derive neurotrophic factor; IGF‐1, insulin‐like growth factor 1; JAK, Janus kinase; MALAT1, metastasis‐associated lung adenocarcinoma transcript 1; MMP, matrix metalloproteinase; NGF, nerve growth factors; PDGF‐BB, platelet‐derived growth factor‐BB; PRP, platelet‐rich plasma; SFI, sciatic functional index; STAT, signal transducer and activator of transcription; TGF‐β1, transforming growth factor‐β1; VEGF, vascular endothelial cell growth factor; YAP, yes‐associated protein.

^a^ Genotypic effects are shown in italics.

^b^ Exosomes were embedded in a hydrogel preparation to achieve a slow, steady rate of release.

^c^ Exosomes were delivered using ultrasound‐assisted microbubble destruction.

**TABLE 2:** Investigations of PRP‐derived exosomes for hair growth and skin aging.

Study	Sample source	Target	Phenotypic/Genotypic effects^a^	Activation	Content
*Hair growth*					
Nilforoushzadeh 2020 [21]	Human PL (cord blood)	DPCs	No or minimal change in cell proliferation and migration No or minimal change in hair follicle inductivity markers	None	—
Nilforoushzadeh 2021 [22]	Human PRP (cord blood)	DPCs	No or minimal change in cell proliferation No or minimal change in hair follicle inductivity markers	None	—
*Skin Aging*					
Rosmarwati 2023 [23]	Human PL	IRM	Improve collagen deposition Decrease MMP‐1 levels	None	—

Abbreviations: DPC, dermal papilla cell; IRM, intrinsic aging rat model; MMP‐1, metalloproteinase‐1; PL, platelet lysate; PRP, platelet‐rich plasma.

^a^ Genotypic effects are shown in italics.

### 3.1 PRP‐Exo in Wound Healing

A multitude of studies have observed favorable wound healing effects of *PRP‐derived exosomes* using animal models for diabetes (Table 1). In diabetic patients, hyperglycemia is a significant contributor to the development of chronic wounds characterized by prolonged inflammation, reduced angiogenesis, ulcers, and neuropathy [7]; in animal models, a hyperglycemia condition can be induced through diet, followed by a surgical incision for the evaluation of wound healing effects. Rui et al. [19] obtained platelets using the *apheresis* method, which were then activated using *thrombin and calcium gluconate* to stimulate the production of exosomes. After local injection, diabetic mice treated with *PRP‐derived exosomes* exhibited significantly faster wound contraction with complete healing by day 14, which was attributed to the anti‐inflammatory effects of miRNA‐26b‐5p detected in exosomes [19]. Similar results were reported by Chen et al., where an injection of *PRP‐derived exosomes*—with *thrombin and calcium gluconate* activation—led to a faster wound closure rate, enhanced angiogenesis (corroborated by the observation of improved endothelial cell viability, migration, and tube formation capacity), and improved re‐epithelialization [18]. Authors attributed this effect to a signaling lipid, sphinogosine‐1‐phosphate, detected in *PRP‐derived exosomes* that activates the PI3K/Akt pathway leading to the upregulation of the growth factor VEGF‐A [18].

Two studies reported the detection of growth factors in the *PRP‐derived exosomes* cargo (b‐FGF, PDGF, TGF‐β, and VEGF) [25, 29], which may function in proliferating fibroblasts and endothelial cells, regulating the inflammatory immune response, angiogenesis, and tissue remodeling, as well as promoting collagen synthesis and hair growth [10, 33]. Cao et al. [25] treated fibroblasts with *PRP‐derived exosomes* containing growth factors and observed an increase in cell viability and migration, as well as an increase in expression of a PDGF receptor that potentiates the JAK2/STAT3 pathway leading to the attenuation of hyperglycemia‐induced apoptosis. Similar results were observed by Guo et al., demonstrating an increased cell viability and migration of fibroblasts and endothelial cells. The authors also observed an upregulation of the RhoA/YAP pathway that increases the level of the growth factor CTGF, as well as activation of the PI3K/Akt and Erk1/2 pathways that regulate cell proliferation and angiogenesis [29]. Furthermore, both studies demonstrated wound healing effects in animal models following the administration of *PRP‐derived exosomes* in hydrogel preparations [25, 29].

Wound healing occurs in three stages, including (1) hemostasis and inflammation, (2) proliferation, and (3) tissue remodeling [3]. Following the formation of a fibrin matrix enabling the infiltration of immune cells, inflammation occurs whereby leukocytes such as neutrophils and macrophages work to clear cellular debris, defend against bacterial infections, as well as secrete growth factors and pro‐inflammatory cytokines [3]. In persons with chronic wounds, the inflammation phase is dysregulated—reflected by the excess neutrophils and matrix metalloproteinases (MMPs)—which impedes the transition to the proliferation phase [3]. Rui et al. [19] reported the delivery of miRNA‐26b‐5p by *PRP‐derived exosomes* that inhibited MMP‐8, which reduced the number of infiltrating neutrophils forming neutrophil extracellular traps. Similar results were found by Bakadia et al., where a topical application of *PRP‐derived exosomes* in hydrogels led to a reduction in the number of macrophages and neutrophil extracellular traps, as well as the downregulation of MMP‐9 [24]. Chen et al. also reported the downregulation of inflammatory cytokines and pyroptosis in fibroblasts isolated from patients with diabetic foot ulcers, which was attributed to the upregulation of a long non‐coding RNA (MALAT1) and downregulation of miR‐374a‐5p by *PRP‐exosomes* [26].

Two ongoing clinical trials are investigating a *platelet‐derived exosomes* product sourced from the *apheresis* method for the treatment of donor site wounds during skin grafting (NCT04664738) and as a treatment for diabetic foot ulcers (NCT06319287). In a case report, this product was used to treat a cancer patient with nonhealing scalp wounds [32]. Four weekly treatment sessions were initially conducted where exosomes were applied topically mixed with a collagen carrier; however, incomplete wound healing and lack of product retention at the wound site led to the switch to a fibrin carrier for four additional weekly sessions [32]. Complete healing of one wound and a 96% contraction of a second wound were observed at follow‐up; no adverse reactions were reported [32].

### 3.2 PRP‐Derived Exo in Hair Growth and Skin Rejuvenation

The dermal papilla, composed of mesenchymal cells, plays a critical role during hair follicle morphogenesis as a mediator of signaling pathways (e.g., Wnt/β‐catenin pathway) [33]; during the anagen phase of the hair growth cycle, the interaction between dermal papilla cells and the hair bulge stem cells leads to the formation of a new hair fiber [33]. In androgenetic alopecia, hair follicle miniaturization is linked to a shrinking dermal papilla with reduced hair follicle inductivity [34]. Exosomes, through its role as a paracrine regulator, may restore the dermal papilla and induce hair growth [33]. In two studies, the in vitro effects of *PRP‐ or platelet lysate‐derived exosomes* (sourced from *human cord blood*) were investigated in dermal papilla cells and compared against exosomes derived from other sources (Table 2) [21, 22]. Their results indicate that exosomes were less effective in promoting cell proliferation and in inducing the inductivity of dermal papilla cells [21, 22].

Considering that *platelet‐derived EVs including exosomes* represent the majority of EVs detected in blood [35], and that PRP has been recognized as a valuable treatment option for androgenetic alopecia [1], further studies may be warranted to confirm these findings. This should include studies assessing the effects of *PRP‐derived exosomes* from healthy adult donors obtained with or without activation in dermal papilla cells and in ex vivo hair follicle models, with detailed biological characterizations including the cargo.

One hallmark of the aging skin is the collapse of the collagen matrix mediated by MMPs, which creates an unfavorable microenvironment that compromises the function of fibroblasts [36]. Without stretch or mechanical tension, fragmented collagen causes fibroblast collapse, resulting in reduced collagen production and increased levels of MMPs degrading collagens [36]; this chain of events generates a negative feedback loop that furthers the aging process. Thus, treatments that induce new collagen production can aid in restoring fibroblast function, thereby attenuating the effects of aging. In a study by Rosmarwati et al. (Table 2) [23], *platelet lysate‐derived exosomes* were delivered by injection to a rat model with features of intrinsic aging. The results showed an increased level of collagen deposition in the dermis with reduced levels of MMP‐1 [23], which supports *PRP‐derived exosomes* as a potential antiaging treatment.

### 3.3 Technical Challenges

**FIGURE 2:**
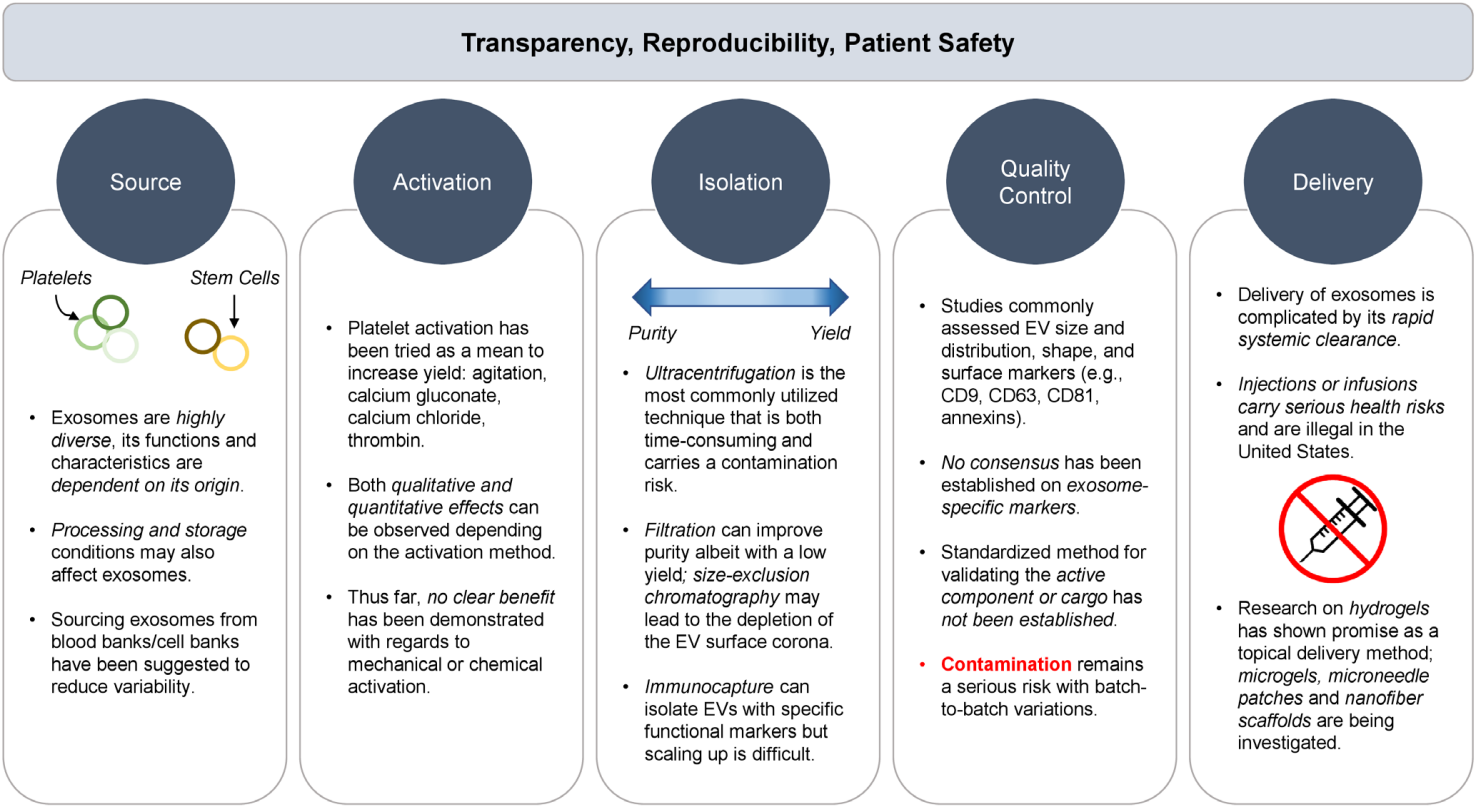
Clinical translation challenges of PRP‐derived exosomes.

Limitations to the use of *PRP‐derived exosomes* include donor‐to‐donor variabilities, as well as non‐standardized activation methods and isolation protocols, that will impact the exosome cargo with varying therapeutic effects [9]. For instance, exosomes isolated from diabetic subjects exhibit qualitative differences from those of healthy donors [37]; PRP obtained from older individuals also appeared less effective [38]. What exactly defines an “active” component of exosomes or cargoes (i.e., proteins, genetic materials) responsible for the genotypic/phenotypic changes in recipient cells remains unclear [39]. In order to establish standards for quality control, the exosome content, in addition to its quantity and size, should be validated. As well, contamination of exosomes with plasma lipoproteins and viruses due to similar sizes is another concern [9]. An overview of the current obstacles in the clinical translation of exosomes is presented in Figure 2.

ISEV has recently published their position on the minimal information recommended for reporting of EV studies [16]. Exosomes represent a subset of EVs with diameters < 200 nm; however, the all‐encompassing term “EV” is preferred over “exosomes” unless an endosomal subcellular origin is proven [16]. Reporting recommendations include descriptions and characterizations of the sample source, storage conditions pre‐ and post‐isolation, assessments of the depletion of cells or other co‐isolates, and implementation of quality controls [16]. For blood‐derived EVs, it is recommended to report donor characteristics, as well as detailing the collection and processing procedures, including the use of anticoagulants and centrifugation settings [16]. Special considerations include the removal of blood cells by centrifugation while preventing hemolysis and the removal of lipoproteins and other soluble proteins that can be co‐isolated with EVs. If samples were pooled from multiple donors, then the number of individual samples should be reported along with matching demographic information and volumes [16]. For studies utilizing ultracentrifugation, details such as the type of rotor and tube, speed, time, temperature, as well as acceleration/deceleration settings should be reported [16]. Recommended EV characterizations, include quantity, size, morphology, protein components, and detection of co‐isolates [16]. In Japan, the Science Board of the Pharmaceuticals and Medical Devices Agency (PMDA) published a review on the quality and safety standards of EVs [39]. To minimize variabilities across studies, the establishment of cell banks was suggested alongside quality control measures for contamination sources. For upscaling exosome production, ultrafiltration, column chromatography, or a combination method may be preferred over ultracentrifugation [39]. In cases where an active exosome component has been identified, an antibody‐based affinity purification/immunocapture method may be considered [39]. Lastly, a public repository for EV research protocols has been established to improve transparency and facilitate standardization (EV‐TRACK) [40].

The EV‐Checklist was used to assess the quality of reporting of the included preclinical studies (see Supporting Information) [17]. In studies where PRP was obtained from human donors, donor characteristics (e.g., medical histories) were frequently not reported. Although freeze–thaw cycles may affect the characteristics of exosomes [16], the thawing procedure, after storing EV preparations at −80°C, was not reported. Quality control measures such as the detection of hemolysis during blood separation or detection of co‐isolates, including lipoproteins or viruses, were also not reported. Nine studies did not report the number of particles (exosomes/mL) [18, 20, 21, 22, 25, 26, 27, 29, 30]. One study did not report methods for exosome isolation and characterization [20]. For isolation (Table S1), most studies employed combinational methods of ultracentrifugation with ultrafiltration (e.g., a separate filtration step following ultracentrifugation or ultracentrifugation with a filtered unit). Two studies added a precipitation step [26, 31], two studies utilized size‐exclusion chromatography [23, 28], and one study utilized tangential‐flow filtration [28].

Although ultracentrifugation is commonly employed for the isolation of exosomes, this method can be limited due to time and sample volume requirements and contamination risk [10]. Thus, the combined use of size‐exclusion chromatography or filtration methods has been suggested to improve the isolation of exosomes [10]. In a study by Gnomes et al. (Table 1) [28], a comparison was made between two fractions of *CaCl*_2_ *‐activated*, *platelet lysate‐derived EVs* that were isolated using either tangential‐flow filtration alone or tangential‐flow filtration followed by size‐exclusion chromatography. When examining the ability of *platelet‐derived EVs* in aiding the formation of human skin organoids, the filtrated fraction—without undergoing size‐exclusion chromatography—was found to be significantly more effective [28]; this fraction was also more effective in inducing fibroblast migration and inhibiting T‐cells. The authors have attributed this difference to the depletion of the EV surface corona by the size‐exclusion chromatography method [28], which are plasma proteins adsorbed around EV surfaces that partially determine its biological activity (Figure 1) [41]. In the absence of the protein corona, *platelet‐derived EVs* exhibited a higher net negative surface charge, affecting their stability and function; re‐introducing the protein corona allowed for the partial recovery of their functions [28].

Activation of platelets leading to the stimulated release of exosomes can be achieved through mechanical means such as agitation causing membrane disruption, as well as by the addition of calcium salts, calcium ionophores, or thrombin that increases exosome secretion through the accumulation of calcium ions in the cytoplasmic space [9]. A study comparing activation methods found a significantly higher quantity of exosomes released from PRP using *calcium gluconate* (23.4 ± 2.9 × 10^8^/mL) compared to *thrombin* (2.1 ± 0.7 × 10^8^/mL) [42]. Besides increasing the quantity, activation methods may also alter the functional profile of exosomes; for instance, Saumell‐Esnaola et al. found that *platelet‐derived exosomes* activated by *CaCl*_*2*_ exhibited a different protein signature than exosomes without activation [43], whereas Goetzl et al. [44] reported an increased level of chemokines and high‐mobility group box 1 in *platelet‐derived exosomes* following *thrombin* activation. To date, no consensus has been established on the benefits of using activation methods. For wound healing (Table 1), we identified varying activation methods utilized across studies (i.e., *thrombin and calcium gluconate*, *thrombin and CaCl*_*2*_, *CaCl*_*2*_ *and Calcimycin*) with positive effects; however, similar results were also reported in studies where no activation methods were used [29, 30]. In contrast, none of the studies assessing hair growth and skin aging employed an activation method (Table 2).

Lastly, exosomes delivered through injections are subjected to a high rate of clearance, limiting their therapeutic effects; hence novel application methods such as embedding exosomes in hydrogels have been investigated [7]. Hydrogels are a network of polymers that expand in volume upon contact with water, with adjustable features that can mimic the conditions of an extracellular matrix [28]. This material can be administered either topically or by injection to improve the pharmacokinetics of exosomes; moreover, some hydrogel preparations can augment the effects of exosomes through wound healing effects [25], antibacterial properties [24], promoting cell proliferation [30], and reducing the levels of reactive oxygen species [7]. Bakadia et al. developed a silk protein‐based hydrogel that delivered *PRP‐derived exosomes* through a controlled, sustained rate of release rather than in bursts (Table 1) [24]; this preparation, either alone or in combination with exosomes, also demonstrated inhibitory activities on bacterial growth [24]. Similar results were observed in other studies applying *PRP‐derived exosomes* topically using hydrogels (Table 1) [29, 30], this opens the door to eliminating the need for local injections, which could help address current safety concerns. Other methods for exosome delivery being investigated include microgels, microneedle patches, and nanofiber scaffolds [7]. These applications could be extended further to investigate the effects of *PRP‐derived exosomes* in hair growth and skin aging.

### 3.4 Regulatory Perspectives and Concerns

Exosomes remain an investigational treatment that is classified as a biologic drug overseen by the Center for Biologics Evaluation and Research (CBER) as part of the U.S. FDA [13, 14], which categorizes exosome products into naïve exosomes (e.g., *PRP‐derived exosomes*) from unmodified or modified sources, modified exosomes from genetically manipulated sources, and naïve exosomes from unmodified sources with synthetic content [13]. The EMA regulates exosomes depending on their cargo [14]. In case if the cargo has a gene regulation effect (e.g., miRNA), then the exosome product will be given the Genetic Therapy Medicine Products (GTMP) designation by the Committee for Advanced Therapies (CAT), otherwise, it will be classified as biologics [14].

During the global SARS‐CoV‐2 pandemic, an uptick in commercial activities making unsubstantiated claims about exosome products—mostly concentrated in the United States and Mexico targeting individuals with long COVID symptoms—was reported [45]. The finding includes businesses offering exosomes via intravenous injections, often marketed directly to consumers on social media platforms, with costs ranging from $4000 to $15 000 [45]. This trend raises serious concerns about patient safety and well‐being, prompting responses from national regulatory bodies.

The U.S. FDA has issued a consumer alert [46], as well as warning letters to businesses (7 warning letters as of November 2024), related to exosome products. Notable violations include a contaminated facility with a lack of good laboratory practice/aseptic techniques resulting in serious adverse events and bacterial infections [47], as well as the marketing of an IND exosome product without a biologics license [48]. Manufacturing of exosomes should adhere to the tenets of the Pharmaceutical Inspection Convention and Current Good Manufacturing Practices (cGMP) or Cooperation Scheme Good Manufacturing Practices (PIC/S GMP) [14]. In the United States, clinical investigations of exosomes have now been restricted to topical administrations only [49]; injections or infusions, either intradermal, intramuscular, or subcutaneous, are illegal [49].

## 4 Conclusions

As opposed to the current use of autologous PRP, *exosomes derived from allogeneic PRP* pooled from healthy donors with well‐defined biological characteristics have the potential to limit interstudy variabilities and may allow for a clearer path to clinical validation. Exosomes are considered as biologics and are currently not approved by any regulatory body worldwide. There is ample preclinical evidence on the use of *PRP‐derived exosomes* for wound healing; however, despite the wide use of PRP among dermatologists, evidence is scarce on the application of *PRP‐derived exosomes* in hair growth and skin rejuvenation. Given the ongoing challenges encompassing the nomenclature, isolation and purification methods, quality control, and clinical translation, further research on exosomes should be centered on transparency, reproducibility, and patient safety. Healthcare providers are cautioned against administering exosomes until more research is done and a clear regulatory framework is developed.

## Author Contributions

Conceptualization, A.K.G., J.A.R., M.T. investigation, T.W. methodology, T.W. resources, A.K.G. visualization, T.W. writing – original draft preparation, T.W. writing – review and editing, A.K.G., J.A.R., M.T.

## Ethics Statement

The authors have nothing to report.

## Conflicts of Interest

The authors declare no conflicts of interest.

## Supporting information


Data S1.

Data S2.

## Data Availability

Data sharing not applicable to this article as no datasets were generated or analyzed during the current study.
