# Supplementary material for: Therapeutic Potential of Extracellular Vesicles (Exosomes) Derived From Platelet‐Rich Plasma: A Literature Review
Source: J Cosmet Dermatol. 2024 Dec 1;24(2):e16709. doi: 10.1111/jocd.16709 (PMC11845942; doi:10.1111/jocd.16709)
Supplement: Supplementary file 1 — Data S1. [file JOCD-24-e16709-s002.pdf]

General information	
Title	Engineering homologous platelet-rich plasma, platelet-rich plasma-derived exosomes, and mesenchymal stem cell-derived exosomes-based dual-crosslinked hydrogels as bioactive diabetic wound dressings
Nomenclature used	Exosomes
Application	Therapeutic
EV-track ID	

Collection and storage		
Releasing cell information	Cell type origin	Animalia / / Blood (Plasma/Serum) /
	Passaging	
	Seeding density	cells/cm²
	Cell viability	
	Culture volume	ml
	Culture vessel	
	Oxygen level	%
Culture conditions	Culturing medium	
	Duration of cultivation	
	Harvesting medium	
	Cell count at harvest	cells
Storage and recovery	Condition medium	°C
	EV preparations	-80 °C

EV isolation		
**Category**	**Methods used**	**Details**
Density based	Ultracentrifugation	At 4 °C: 300 g for 10 minutes, 1500 g for 15 minutes, 3200 g for 20 minutes, then 20,000 g for 30 minutes.<br><br>After ultrafiltration: 100,000 g for 70 minutes at 4 °C, wash with PBS, then pelleted again under the same conditions and reconstituted in PBS.<br><br>Using Nanosep centrifugal device: 14,000 g for 20 minutes at 4 °C
Size based	size exclusion	Ultrafiltration with a 0.22 µm filter.<br>After reconstituion: Nanosep centrifugal device at 100 kDa.
Precipitation based	/	/
Affinity purification	/	/

EV isolation		
**Category**	**Methods used**	**Details**
Chromatography	/	/
Microfluidics	/	/

EV characterization				
		**Parameter**	**Unit**	**Method**
Quantification	Size & concentration	Particle number	6.94x10e10 particles/ml	Nano tracking analysis
		Particle size	60 ± 10 nm	Nano tracking analysis
		Particle/protein ratio	particles/µg	
	Composition	Protein content	/	/
		Lipid content	/	/
		RNA content	/	/
Identity		Category 1- Transmembrane or GPI-anchored proteins associated to plasma membrane and/or endosomes	CD63;CD81;CD9	Western blot,
		Category 2- Cytosolic proteins recovered in EVs	/	Western blot,
		Category 3- Major components of non-EV co-isolated structures	/	Western blot,
Visualization		TEM	TEM,	

EV function(s)	
Vasculogenesis and Angiogenesis;Immunomodulation;Endogenous regeneration	

MiBlood-EV		
Blood study information	Time period of experiment (Years)	/
	Number of samples	32
	Biospecimen type	Plasma
	Biospecimen state	Fresh
	Source of frozen specimen	Prepared by the lab
	Patient fasting status	Uncertain
	Fasting length (e.g. hours/days)	N/A

MiBlood-EV		
Blood collection and processing	Anatomical access site	Heart
	Needle diameter (e.g. gauge)	20
	Blood volume collected (mL)	N/A
	Plasma anticoagulant	EDTA
	Other plasma anticoagulant	/
	Serum tube type	/
	Serum clotting time (minutes)	/
	Time between collection and first centrifugation (range in hours)	/
	Transport temperature	/
	Transport condition of tubes	/
	Centrifuge brand and model	/
	Bucket rotor type	/
	Number of centrifugation cycles	2
	1st Centrifugation speed (RCF in x g)	900
	1st Rotor brake	/
	1st Centrifugation temperature	/
	2nd Centrifugation speed (RCF in x g)	1500
	2nd Rotor brake	/
	2nd Centrifugation temperature	/
	Additional processing steps (e.g. filtration)	Addition of prostaglandin E1 (100 ng/ml) to prevent platelet activation before the second centrifugation step.
	Storage tubes (brand, type, source, catalog number)	/
	Storage temperature	Uncertain
	Length of storage (range in years)	/
	Number of freeze-thaw cycles (range)	/
	Thawing temperature (°C)	/
	Thawing duration (minutes)	/
	Presence of hemolysis	Not tested
	Number of samples affected (e.g. <25%, 25-50%)	/
	Method used	/
	Other method used	/
	RBC count (Median, 95% CI, N)	/

MiBlood-EV		
Plasma/Serum quality control	RBC counter brand and type	/
	Spectrophotometry hemoglobin concentration (g/L)	/
	Spectrophotometer brand, model and wavelength measured (e.g. 414 nm)	/
	Hemolized samples were discarded	/
	Presence of platelets	Yes
	Method used for platelets	/
	Other method used	/
	Platelet marker(s) used (e.g. CD61, CD41)	/
	Concentration (median, 95% CI, N)	/
	Platelet counter instrument brand and type	/
	Flow cytometer brand and type	/
	Flow cytometry size and fluorescence ranges of detection in nanometers and MESF, respectively	/
	Presence of lipoproteins	/
	Method used (WB, ELISA, FC)	/
	Other method used	/
	Spectrophotometry L-index	/
	Spectrophotometer brand, model and wavelength measured (e.g. 700 nm)	/
	WB Marker(s) used (e.g. Apo B)	/
	Western blot images provided in manuscript?	/
	Flow cytometry marker(s) used (e.g. ApoB)	/
	Flow cytometry concentration (median, 95% CI, N)	/
	Flow cytometer brand and type	/
	Flow cytometry size and fluorescence ranges of detection in nanometers and MESF, respectively	/

Other information

General information	
Title	Exosomes derived from platelet-rich plasma promote diabetic wound healing via the JAK2/ STAT3 pathway
Nomenclature used	Exosomes
Application	Therapeutic
EV-track ID	

Collection and storage		
Releasing cell information	Cell type origin	Animalia / / Blood (Plasma/Serum) /
	Passaging	
	Seeding density	cells/cm²
	Cell viability	
	Culture volume	ml
	Culture vessel	
	Oxygen level	%
Culture conditions	Culturing medium	
	Duration of cultivation	
	Harvesting medium	
	Cell count at harvest	cells
Storage and recovery	Condition medium	°C
	EV preparations	-80 °C

EV isolation		
**Category**	**Methods used**	**Details**
Density based	Ultracentrifugation	250 g x 15 minutes, 300 g x 10 minutes, 2000 g x 10 minutes, ultrafiltration, 4000 g x 50 minutes (15ml ultrafiltration centrifugal tube), wash with PBS, 400 g x 50 minutes (15ml ultrafiltration centrifugal tube), 100,000 g x 70 minutes, wash with PBS, 100,000 g x 70 minutes
Size based	size exclusion	Ultrafiltration with a 0.22 µm filter
Precipitation based	/	/
Affinity purification	/	/
Chromatography	/	/
Microfluidics	/	/

EV characterization				
		**Parameter**	**Unit**	**Method**
Quantification	Size & concentration	Particle number	/	/
		Particle size	117.5 nm	Nano tracking analysis
		Particle/protein ratio	/	/
	Composition	Protein content	/	/
		Lipid content	/	/
		RNA content	/	/
Identity		Category 1- Transmembrane or GPI-anchored proteins associated to plasma membrane and/or endosomes	CD41;CD63;CD81;CD9	Western blot,
		Category 2- Cytosolic proteins recovered in EVs	/	Western blot,
		Category 3- Major components of non-EV co-isolated structures	/	Western blot,
Visualization		TEM	TEM,	

EV function(s)	
Cytoprotection;Vasculogenesis and Angiogenesis;Endogenous regeneration	

MiBlood-EV		
Blood study information	Time period of experiment (Years)	/
	Number of samples	/
	Biospecimen type	Plasma
	Biospecimen state	Fresh
	Source of frozen specimen	/
	Patient fasting status	Uncertain
	Fasting length (e.g. hours/days)	/
	Anatomical access site	/
	Needle diameter (e.g. gauge)	/
	Blood volume collected (mL)	/
	Plasma anticoagulant	/
	Other plasma anticoagulant	ACD-A
	Serum tube type	/

MiBlood-EV		
Blood collection and processing	Serum clotting time (minutes)	/
	Time between collection and first centrifugation (range in hours)	/
	Transport temperature	/
	Transport condition of tubes	/
	Centrifuge brand and model	/
	Bucket rotor type	/
	Number of centrifugation cycles	/
	1st Centrifugation speed (RCF in x g)	160
	1st Rotor brake	/
	1st Centrifugation temperature	/
	2nd Centrifugation speed (RCF in x g)	250
	2nd Rotor brake	/
	2nd Centrifugation temperature	/
	Additional processing steps (e.g. filtration)	/
	Storage tubes (brand, type, source, catalog number)	/
	Storage temperature	/
	Length of storage (range in years)	/
	Number of freeze-thaw cycles (range)	/
	Thawing temperature (°C)	/
	Thawing duration (minutes)	/
	Presence of hemolysis	Not tested
	Number of samples affected (e.g. <25%, 25-50%)	/
	Method used	/
	Other method used	/
	RBC count (Median, 95% CI, N)	/
	RBC counter brand and type	/
	Spectrophotometry hemoglobin concentration (g/L)	/
	Spectrophotometer brand, model and wavelength measured (e.g. 414 nm)	/
	Hemolized samples were discarded	/
	Presence of platelets	Yes
	Method used for platelets	/

MiBlood-EV		
Plasma/Serum quality control	Other method used	/
	Platelet marker(s) used (e.g. CD61, CD41)	/
	Concentration (median, 95% CI, N)	/
	Platelet counter instrument brand and type	/
	Flow cytometer brand and type	/
	Flow cytometry size and fluorescence ranges of detection in nanometers and MESF, respectively	/
	Presence of lipoproteins	/
	Method used (WB, ELISA, FC)	/
	Other method used	/
	Spectrophotometry L-index	/
	Spectrophotometer brand, model and wavelength measured (e.g. 700 nm)	/
	WB Marker(s) used (e.g. Apo B)	/
	Western blot images provided in manuscript?	/
	Flow cytometry marker(s) used (e.g. ApoB)	/
	Flow cytometry concentration (median, 95% CI, N)	/
	Flow cytometer brand and type	/
	Flow cytometry size and fluorescence ranges of detection in nanometers and MESF, respectively	/

Other information

General information	
Title	Sphingosine-1-phosphate derived from PRP-Exos promotes angiogenesis in diabetic wound healing via the S1PR1/AKT/FN1 signalling pathway
Nomenclature used	Exosomes
Application	Therapeutic
EV-track ID	

Collection and storage		
Releasing cell information	Cell type origin	Animalia / / Blood (Plasma/Serum) /
	Passaging	
	Seeding density	cells/cm²
	Cell viability	
	Culture volume	ml
	Culture vessel	
	Oxygen level	%
Culture conditions	Culturing medium	
	Duration of cultivation	
	Harvesting medium	
	Cell count at harvest	cells
Storage and recovery	Condition medium	°C
	EV preparations	-80 °C

EV isolation		
**Category**	**Methods used**	**Details**
Density based	Ultracentrifugation	500 g x 10 minutes, 1500 g x 10 minutes, 2500 g x 10 minutes, filtration, 100,000 g x 70 minutes repeated twice, PBS wash, 100,000 g x 70 minutes
Size based	size exclusion	Filtration to remove large residues: 0.45 μm filter
Precipitation based	/	/
Affinity purification	/	/
Chromatography	/	/
Microfluidics	/	/

EV characterization				
		Parameter	Unit	Method
Quantification	Size & concentration	Particle number	/	/
		Particle size	50-150 nm	Nano tracking analysis
		Particle/protein ratio	/	/
	Composition	Protein content	/	/
		Lipid content	/	/
		RNA content	/	/
Identity		Category 1- Transmembrane or GPI-anchored proteins associated to plasma membrane and/or endosomes	CD41;CD63	/,
		Category 2- Cytosolic proteins recovered in EVs	Flotillin;TSG101	/,
		Category 3- Major components of non-EV co-isolated structures	/	/,
Visualization		TEM	TEM,	

EV function(s)	
Vasculogenesis and Angiogenesis;Endogenous regeneration	

MiBlood-EV		
Blood study information	Time period of experiment (Years)	/
	Number of samples	15
	Biospecimen type	Plasma
	Biospecimen state	Fresh
	Source of frozen specimen	/
	Patient fasting status	/
	Fasting length (e.g. hours/days)	/
	Anatomical access site	/
	Needle diameter (e.g. gauge)	/
	Blood volume collected (mL)	/
	Plasma anticoagulant	/
	Other plasma anticoagulant	/
	Serum tube type	/

MiBlood-EV		
Blood collection and processing	Serum clotting time (minutes)	/
	Time between collection and first centrifugation (range in hours)	/
	Transport temperature	/
	Transport condition of tubes	/
	Centrifuge brand and model	/
	Bucket rotor type	/
	Number of centrifugation cycles	/
	1st Centrifugation speed (RCF in x g)	/
	1st Rotor brake	/
	1st Centrifugation temperature	/
	2nd Centrifugation speed (RCF in x g)	/
	2nd Rotor brake	/
	2nd Centrifugation temperature	/
	Additional processing steps (e.g. filtration)	/
	Storage tubes (brand, type, source, catalog number)	/
	Storage temperature	/
	Length of storage (range in years)	/
	Number of freeze-thaw cycles (range)	/
	Thawing temperature (°C)	/
	Thawing duration (minutes)	/
	Presence of hemolysis	Not tested
	Number of samples affected (e.g. <25%, 25-50%)	/
	Method used	/
	Other method used	/
	RBC count (Median, 95% CI, N)	/
	RBC counter brand and type	/
	Spectrophotometry hemoglobin concentration (g/L)	/
	Spectrophotometer brand, model and wavelength measured (e.g. 414 nm)	/
	Hemolized samples were discarded	/
	Presence of platelets	/
	Method used for platelets	/

MiBlood-EV		
Plasma/Serum quality control	Other method used	/
	Platelet marker(s) used (e.g. CD61, CD41)	/
	Concentration (median, 95% CI, N)	/
	Platelet counter instrument brand and type	/
	Flow cytometer brand and type	/
	Flow cytometry size and fluorescence ranges of detection in nanometers and MESF, respectively	/
	Presence of lipoproteins	/
	Method used (WB, ELISA, FC)	/
	Other method used	/
	Spectrophotometry L-index	/
	Spectrophotometer brand, model and wavelength measured (e.g. 700 nm)	/
	WB Marker(s) used (e.g. Apo B)	/
	Western blot images provided in manuscript?	/
	Flow cytometry marker(s) used (e.g. ApoB)	/
	Flow cytometry concentration (median, 95% CI, N)	/
	Flow cytometer brand and type	/
	Flow cytometry size and fluorescence ranges of detection in nanometers and MESF, respectively	/

Other information

General information	
Title	MALAT1 participates in the role of platelet-rich plasma exosomes in promoting wound healing of diabetic foot ulcer
Nomenclature used	Exosomes
Application	Therapeutic
EV-track ID	

Collection and storage		
Releasing cell information	Cell type origin	Animalia / / Blood (Plasma/Serum) /
	Passaging	
	Seeding density	cells/cm²
	Cell viability	
	Culture volume	ml
	Culture vessel	
	Oxygen level	%
Culture conditions	Culturing medium	
	Duration of cultivation	
	Harvesting medium	
	Cell count at harvest	cells
Storage and recovery	Condition medium	°C
	EV preparations	-80 °C

EV isolation

Category	Methods used	Details
Density based	Ultracentrifugation	250 g x 15 minutes, 10,000 g x 10 minutes, washed with PBS, add Blood PureExo Solution, 10,000 g x 10 minutes, filtered, 3000 g x 10 minutes
Size based	size exclusion	Exosome Purification Filter
Precipitation based	/	Blood PureExo Solution
Affinity purification	/	/
Chromatography	/	/
Microfluidics	/	/

EV characterization				
		Parameter	Unit	Method
Quantification	Size & concentration	Particle number	/	/
		Particle size	40-100 nm	Nano tracking analysis
		Particle/protein ratio	/	/
	Composition	Protein content	/	/
		Lipid content	/	/
		RNA content	/	/
Identity		Category 1- Transmembrane or GPI-anchored proteins associated to plasma membrane and/or endosomes	/	
		Category 2- Cytosolic proteins recovered in EVs	/	
		Category 3- Major components of non-EV co-isolated structures	/	
Visualization		TEM	TEM,	/

EV function(s)	
Cytoprotection;Immunomodulation;End[illegible] regeneration	

MiBlood-EV		
Blood study information	Time period of experiment (Years)	/
	Number of samples	/
	Biospecimen type	Plasma
	Biospecimen state	Fresh
	Source of frozen specimen	/
	Patient fasting status	/
	Fasting length (e.g. hours/days)	/
	Anatomical access site	/
	Needle diameter (e.g. gauge)	/
	Blood volume collected (mL)	/
	Plasma anticoagulant	/
	Other plasma anticoagulant	ACD-A
	Serum tube type	/
	Serum clotting time (minutes)	/

MiBlood-EV		
Blood collection and processing	Time between collection and first centrifugation (range in hours)	/
	Transport temperature	/
	Transport condition of tubes	/
	Centrifuge brand and model	/
	Bucket rotor type	/
	Number of centrifugation cycles	/
	1st Centrifugation speed (RCF in x g)	160 g
	1st Rotor brake	/
	1st Centrifugation temperature	/
	2nd Centrifugation speed (RCF in x g)	1000 g
	2nd Rotor brake	/
	2nd Centrifugation temperature	/
	Additional processing steps (e.g. filtration)	/
	Storage tubes (brand, type, source, catalog number)	/
	Storage temperature	/
	Length of storage (range in years)	/
	Number of freeze-thaw cycles (range)	/
	Thawing temperature (°C)	/
	Thawing duration (minutes)	/
	Presence of hemolysis	No
	Number of samples affected (e.g. <25%, 25-50%)	/
	Method used	/
	Other method used	/
	RBC count (Median, 95% CI, N)	/
	RBC counter brand and type	/
	Spectrophotometry hemoglobin concentration (g/L)	/
	Spectrophotometer brand, model and wavelength measured (e.g. 414 nm)	/
	Hemolized samples were discarded	/
	Presence of platelets	/
	Method used for platelets	/
	Other method used	/

MiBlood-EV		
Plasma/Serum quality control	Platelet marker(s) used (e.g. CD61, CD41)	/
	Concentration (median, 95% CI, N)	/
	Platelet counter instrument brand and type	/
	Flow cytometer brand and type	/
	Flow cytometry size and fluorescence ranges of detection in nanometers and MESF, respectively	/
	Presence of lipoproteins	/
	Method used (WB, ELISA, FC)	/
	Other method used	/
	Spectrophotometry L-index	/
	Spectrophotometer brand, model and wavelength measured (e.g. 700 nm)	/
	WB Marker(s) used (e.g. Apo B)	/
	Western blot images provided in manuscript?	/
	Flow cytometry marker(s) used (e.g. ApoB)	/
	Flow cytometry concentration (median, 95% CI, N)	/
	Flow cytometer brand and type	/
	Flow cytometry size and fluorescence ranges of detection in nanometers and MESF, respectively	/

Other information

General information	
Title	Synergy of Human Platelet-Derived Extracellular Vesicles with Secretome Proteins Promotes Regenerative Functions
Nomenclature used	Extracellular vesicles (EV)
Application	Therapeutic
EV-track ID	

Collection and storage		
Releasing cell information	Cell type origin	Animalia / / Platelets /
	Passaging	
	Seeding density	cells/cm²
	Cell viability	
	Culture volume	ml
	Culture vessel	
	Oxygen level	%
Culture conditions	Culturing medium	
	Duration of cultivation	
	Harvesting medium	
	Cell count at harvest	cells
Storage and recovery	Condition medium	°C
	EV preparations	N/A °C

EV isolation		
**Category**	**Methods used**	**Details**
Density based	/	/
Size based	size exclusion;tangential flow filtration	0.22 µm filter, then tangential flow filtration
Precipitation based	/	/
Affinity purification	/	/
Chromatography	NA	qEV2 size-exclusion chromatography (Izon Science, Christ Church, New Zealand)
Microfluidics	/	/

<table>
<tr><th colspan="5">EV characterization</th></tr>
<tr><th colspan="2"></th><th>Parameter</th><th>Unit</th><th>Method</th></tr>
<tr><td rowspan="6">Quantification</td><td rowspan="3">Size & concentration</td><td>Particle number</td><td>2.2x10e12 particles/ml</td><td>/</td></tr>
<tr><td>Particle size</td><td>/</td><td>/</td></tr>
<tr><td>Particle/protein ratio</td><td>particles/µg</td><td></td></tr>
<tr><td rowspan="3">Composition</td><td>Protein content</td><td>132.3 (SD: 16.1) mg/ml</td><td>/</td></tr>
<tr><td>Lipid content</td><td>/</td><td>/</td></tr>
<tr><td>RNA content</td><td>/</td><td>/</td></tr>
<tr><td colspan="2" rowspan="3">Identity</td><td>Category 1- Transmembrane or GPI-anchored proteins associated to plasma membrane and/or endosomes</td><td>CD63;CD9</td><td>/,</td></tr>
<tr><td>Category 2- Cytosolic proteins recovered in EVs</td><td>/</td><td>/,</td></tr>
<tr><td>Category 3- Major components of non-EV co-isolated structures</td><td>/</td><td>/,</td></tr>
<tr><td colspan="2">Visualization</td><td colspan="2">/</td><td>/,</td></tr>
</table>

<table>
<tr><th colspan="2">EV function(s)</th></tr>
<tr><td>Vasculogenesis and Angiogenesis;Endogenous regeneration</td><td></td></tr>
</table>

Other information

General information	
Title	Exosomes derived from platelet-rich plasma promote the re-epithelization of chronic cutaneous wounds via activation of YAP in a diabetic rat model
Nomenclature used	Exosomes
Application	Therapeutic
EV-track ID	

Collection and storage		
Releasing cell information	Cell type origin	Animalia / / Blood (Plasma/Serum) /
	Passaging	
	Seeding density	cells/cm²
	Cell viability	
	Culture volume	ml
	Culture vessel	
	Oxygen level	%
Culture conditions	Culturing medium	
	Duration of cultivation	
	Harvesting medium	
	Cell count at harvest	cells
Storage and recovery	Condition medium	°C
	EV preparations	-80 °C

EV isolation

Category	Methods used	Details
Density based	Ultracentrifugation	250 g x 15 minutes, wash 3x with PBS, 300 g x 10 minutes, 2000 g x 10 minutes, ultrafiltration, 4000 g using the a filter unit, wash 3x with PBS, 4000 g, 100,000 g x 70 minutes
Size based	Ultrafiltration	0.22 µm filter<br>15 mL Amicon Ultra-15 Centrifugal Filter Unit
Precipitation based	/	/
Affinity purification	/	/
Chromatography	/	/
Microfluidics	/	/

EV characterization				
		Parameter	Unit	Method
Quantification	Size & concentration	Particle number	/	/
		Particle size	40-100 nm	/
		Particle/protein ratio	/	/
	Composition	Protein content	/	/
		Lipid content	/	/
		RNA content	/	/
Identity		Category 1- Transmembrane or GPI-anchored proteins associated to plasma membrane and/or endosomes	CD41;CD63;CD81;CD9	Western blot,
		Category 2- Cytosolic proteins recovered in EVs	/	Western blot,
		Category 3- Major components of non-EV co-isolated structures	/	Western blot,
Visualization		TEM	TEM,	

EV function(s)	
Vasculogenesis and Angiogenesis;Endogenous regeneration	

MiBlood-EV		
Blood study information	Time period of experiment (Years)	/
	Number of samples	/
	Biospecimen type	Plasma
	Biospecimen state	Fresh
	Source of frozen specimen	/
	Patient fasting status	/
	Fasting length (e.g. hours/days)	/
	Anatomical access site	/
	Needle diameter (e.g. gauge)	/
	Blood volume collected (mL)	/
	Plasma anticoagulant	/
	Other plasma anticoagulant	ACD-A
	Serum tube type	/

MiBlood-EV		
Blood collection and processing	Serum clotting time (minutes)	/
	Time between collection and first centrifugation (range in hours)	/
	Transport temperature	/
	Transport condition of tubes	/
	Centrifuge brand and model	/
	Bucket rotor type	/
	Number of centrifugation cycles	/
	1st Centrifugation speed (RCF in x g)	160
	1st Rotor brake	/
	1st Centrifugation temperature	/
	2nd Centrifugation speed (RCF in x g)	250
	2nd Rotor brake	/
	2nd Centrifugation temperature	/
	Additional processing steps (e.g. filtration)	/
	Storage tubes (brand, type, source, catalog number)	/
	Storage temperature	/
	Length of storage (range in years)	/
	Number of freeze-thaw cycles (range)	/
	Thawing temperature (°C)	/
	Thawing duration (minutes)	/
	Presence of hemolysis	/
	Number of samples affected (e.g. <25%, 25-50%)	/
	Method used	/
	Other method used	/
	RBC count (Median, 95% CI, N)	/
	RBC counter brand and type	/
	Spectrophotometry hemoglobin concentration (g/L)	/
	Spectrophotometer brand, model and wavelength measured (e.g. 414 nm)	/
	Hemolized samples were discarded	/
	Presence of platelets	/
	Method used for platelets	/

MiBlood-EV		
Plasma/Serum quality control	Other method used	/
	Platelet marker(s) used (e.g. CD61, CD41)	/
	Concentration (median, 95% CI, N)	/
	Platelet counter instrument brand and type	/
	Flow cytometer brand and type	/
	Flow cytometry size and fluorescence ranges of detection in nanometers and MESF, respectively	/
	Presence of lipoproteins	/
	Method used (WB, ELISA, FC)	/
	Other method used	/
	Spectrophotometry L-index	/
	Spectrophotometer brand, model and wavelength measured (e.g. 700 nm)	/
	WB Marker(s) used (e.g. Apo B)	/
	Western blot images provided in manuscript?	/
	Flow cytometry marker(s) used (e.g. ApoB)	/
	Flow cytometry concentration (median, 95% CI, N)	/
	Flow cytometer brand and type	/
	Flow cytometry size and fluorescence ranges of detection in nanometers and MESF, respectively	/

Other information

General information	
Title	Platelet rich plasma-derived microvesicles increased in vitro wound healing
Nomenclature used	Microvesicles (MV)
Application	Therapeutic
EV-track ID	

Collection and storage		
Releasing cell information	Cell type origin	Animalia / / Blood (Plasma/Serum) /
	Passaging	
	Seeding density	cells/cm²
	Cell viability	
	Culture volume	ml
	Culture vessel	
	Oxygen level	%
Culture conditions	Culturing medium	
	Duration of cultivation	
	Harvesting medium	
	Cell count at harvest	cells
Storage and recovery	Condition medium	°C
	EV preparations	N/A °C

EV isolation

Category	Methods used	Details
Density based	Ultracentrifugation	20000 g x 40 minutes
Size based	/	/
Precipitation based	/	/
Affinity purification	/	/
Chromatography	/	/
Microfluidics	/	/

EV characterization				
		Parameter	Unit	Method
Quantification	Size & concentration	Particle number	/	/
		Particle size	/	/
		Particle/protein ratio	/	/
	Composition	Protein content	/	/
		Lipid content	/	/
		RNA content	/	/
Identity		Category 1- Transmembrane or GPI-anchored proteins associated to plasma membrane and/or endosomes	/	
		Category 2- Cytosolic proteins recovered in EVs	/	
		Category 3- Major components of non-EV co-isolated structures	/	
Visualization		/		

EV function(s)	
Endogenous regeneration	

MiBlood-EV		
Blood study information	Time period of experiment (Years)	/
	Number of samples	/
	Biospecimen type	Plasma
	Biospecimen state	Fresh
	Source of frozen specimen	/
	Patient fasting status	/
	Fasting length (e.g. hours/days)	/
	Anatomical access site	/
	Needle diameter (e.g. gauge)	/
	Blood volume collected (mL)	/
	Plasma anticoagulant	Heparin
	Other plasma anticoagulant	/
	Serum tube type	/
	Serum clotting time (minutes)	/

MiBlood-EV		
Blood collection and processing	Time between collection and first centrifugation (range in hours)	/
	Transport temperature	/
	Transport condition of tubes	/
	Centrifuge brand and model	/
	Bucket rotor type	/
	Number of centrifugation cycles	/
	1st Centrifugation speed (RCF in x g)	/
	1st Rotor brake	/
	1st Centrifugation temperature	/
	2nd Centrifugation speed (RCF in x g)	/
	2nd Rotor brake	/
	2nd Centrifugation temperature	/
	Additional processing steps (e.g. filtration)	/
	Storage tubes (brand, type, source, catalog number)	/
	Storage temperature	/
	Length of storage (range in years)	/
	Number of freeze-thaw cycles (range)	/
	Thawing temperature (°C)	/
	Thawing duration (minutes)	/
	Presence of hemolysis	/
	Number of samples affected (e.g. <25%, 25-50%)	/
	Method used	/
	Other method used	/
	RBC count (Median, 95% CI, N)	/
	RBC counter brand and type	/
	Spectrophotometry hemoglobin concentration (g/L)	/
	Spectrophotometer brand, model and wavelength measured (e.g. 414 nm)	/
	Hemolized samples were discarded	/
	Presence of platelets	/
	Method used for platelets	/
	Other method used	/

MiBlood-EV		
Plasma/Serum quality control	Platelet marker(s) used (e.g. CD61, CD41)	/
	Concentration (median, 95% CI, N)	/
	Platelet counter instrument brand and type	/
	Flow cytometer brand and type	/
	Flow cytometry size and fluorescence ranges of detection in nanometers and MESF, respectively	/
	Presence of lipoproteins	/
	Method used (WB, ELISA, FC)	/
	Other method used	/
	Spectrophotometry L-index	/
	Spectrophotometer brand, model and wavelength measured (e.g. 700 nm)	/
	WB Marker(s) used (e.g. Apo B)	/
	Western blot images provided in manuscript?	/
	Flow cytometry marker(s) used (e.g. ApoB)	/
	Flow cytometry concentration (median, 95% CI, N)	/
	Flow cytometer brand and type	/
	Flow cytometry size and fluorescence ranges of detection in nanometers and MESF, respectively	/

Other information

General information	
Title	Human Hair Outer Root Sheath Cells and Platelet-Lysis Exosomes Promote Hair Inductivity of Dermal Papilla Cell
Nomenclature used	Exosomes
Application	Therapeutic
EV-track ID	

Collection and storage		
Releasing cell information	Cell type origin	Animalia / / Blood (Plasma/Serum) /
	Passaging	
	Seeding density	cells/cm²
	Cell viability	
	Culture volume	ml
	Culture vessel	
	Oxygen level	%
Culture conditions	Culturing medium	
	Duration of cultivation	
	Harvesting medium	
	Cell count at harvest	cells
Storage and recovery	Condition medium	°C
	EV preparations	-80 °C

EV isolation		
**Category**	**Methods used**	**Details**
Density based	Ultracentrifugation	300 g x 10 minutes, 2000 g x 10 minutes, 10,000 g, 100,000 g for 2 hours
Size based	/	/
Precipitation based	/	/
Affinity purification	/	/
Chromatography	/	/
Microfluidics	/	/

EV characterization				
		Parameter	Unit	Method
Quantification	Size & concentration	Particle number	/	/
		Particle size	30-100 nm	Nano tracking analysis
		Particle/protein ratio	/	/
	Composition	Protein content	/	/
		Lipid content	/	/
		RNA content	/	/
Identity		Category 1- Transmembrane or GPI-anchored proteins associated to plasma membrane and/or endosomes	CD63;CD81;CD9	/,
		Category 2- Cytosolic proteins recovered in EVs	TSG101	/,
		Category 3- Major components of non-EV co-isolated structures	/	/,
Visualization		TEM	TEM,	

EV function(s)	
Endogenous regeneration	

MiBlood-EV		
Blood study information	Time period of experiment (Years)	/
	Number of samples	/
	Biospecimen type	/
	Biospecimen state	Fresh
	Source of frozen specimen	/
	Patient fasting status	/
	Fasting length (e.g. hours/days)	/
	Anatomical access site	/
	Needle diameter (e.g. gauge)	/
	Blood volume collected (mL)	/
	Plasma anticoagulant	/
	Other plasma anticoagulant	/
	Serum tube type	/
	Serum clotting time (minutes)	/

MiBlood-EV		
Blood collection and processing	Time between collection and first centrifugation (range in hours)	/
	Transport temperature	/
	Transport condition of tubes	/
	Centrifuge brand and model	/
	Bucket rotor type	/
	Number of centrifugation cycles	/
	1st Centrifugation speed (RCF in x g)	/
	1st Rotor brake	/
	1st Centrifugation temperature	/
	2nd Centrifugation speed (RCF in x g)	/
	2nd Rotor brake	/
	2nd Centrifugation temperature	/
	Additional processing steps (e.g. filtration)	/
	Storage tubes (brand, type, source, catalog number)	/
	Storage temperature	/
	Length of storage (range in years)	/
	Number of freeze-thaw cycles (range)	/
	Thawing temperature (°C)	/
	Thawing duration (minutes)	/
	Presence of hemolysis	/
	Number of samples affected (e.g. <25%, 25-50%)	/
	Method used	/
	Other method used	/
	RBC count (Median, 95% CI, N)	/
	RBC counter brand and type	/
	Spectrophotometry hemoglobin concentration (g/L)	/
	Spectrophotometer brand, model and wavelength measured (e.g. 414 nm)	/
	Hemolized samples were discarded	/
	Presence of platelets	/
	Method used for platelets	/
	Other method used	/

MiBlood-EV		
Plasma/Serum quality control	Platelet marker(s) used (e.g. CD61, CD41)	/
	Concentration (median, 95% CI, N)	/
	Platelet counter instrument brand and type	/
	Flow cytometer brand and type	/
	Flow cytometry size and fluorescence ranges of detection in nanometers and MESF, respectively	/
	Presence of lipoproteins	/
	Method used (WB, ELISA, FC)	/
	Other method used	/
	Spectrophotometry L-index	/
	Spectrophotometer brand, model and wavelength measured (e.g. 700 nm)	/
	WB Marker(s) used (e.g. Apo B)	/
	Western blot images provided in manuscript?	/
	Flow cytometry marker(s) used (e.g. ApoB)	/
	Flow cytometry concentration (median, 95% CI, N)	/
	Flow cytometer brand and type	/
	Flow cytometry size and fluorescence ranges of detection in nanometers and MESF, respectively	/

Other information

General information	
Title	Effects of Adipose-Derived Stem Cells and Platelet-Rich Plasma Exosomes on The Inductivity of Hair Dermal Papilla Cells
Nomenclature used	Exosomes
Application	Therapeutic
EV-track ID	

Collection and storage		
Releasing cell information	Cell type origin	Animalia / / Blood (Plasma/Serum) /
	Passaging	
	Seeding density	cells/cm²
	Cell viability	
	Culture volume	ml
	Culture vessel	
	Oxygen level	%
Culture conditions	Culturing medium	
	Duration of cultivation	
	Harvesting medium	
	Cell count at harvest	cells
Storage and recovery	Condition medium	°C
	EV preparations	-80 °C

EV isolation

Category	Methods used	Details
Density based	Ultracentrifugation	300 g x 10 minutes, 2000 g x 10 minutes, 10,000 g, 10,000 g x 2 hours repeated twice
Size based	/	/
Precipitation based	/	/
Affinity purification	/	/
Chromatography	/	/
Microfluidics	/	/

EV characterization				
		Parameter	Unit	Method
Quantification	Size & concentration	Particle number	/	/
		Particle size	50-150 nm	Nano tracking analysis
		Particle/protein ratio	/	/
	Composition	Protein content	/	/
		Lipid content	/	/
		RNA content	/	/
Identity		Category 1- Transmembrane or GPI-anchored proteins associated to plasma membrane and/or endosomes	CD63;CD81;CD9	/,
		Category 2- Cytosolic proteins recovered in EVs	TSG101	/,
		Category 3- Major components of non-EV co-isolated structures	/	/,
Visualization		TEM	TEM,	

EV function(s)	
Endogenous regeneration	

MiBlood-EV		
Blood study information	Time period of experiment (Years)	/
	Number of samples	/
	Biospecimen type	/
	Biospecimen state	/
	Source of frozen specimen	/
	Patient fasting status	/
	Fasting length (e.g. hours/days)	/
	Anatomical access site	/
	Needle diameter (e.g. gauge)	/
	Blood volume collected (mL)	/
	Plasma anticoagulant	/
	Other plasma anticoagulant	/
	Serum tube type	/
	Serum clotting time (minutes)	/

MiBlood-EV		
Blood collection and processing	Time between collection and first centrifugation (range in hours)	/
	Transport temperature	/
	Transport condition of tubes	/
	Centrifuge brand and model	/
	Bucket rotor type	/
	Number of centrifugation cycles	/
	1st Centrifugation speed (RCF in x g)	/
	1st Rotor brake	/
	1st Centrifugation temperature	/
	2nd Centrifugation speed (RCF in x g)	/
	2nd Rotor brake	/
	2nd Centrifugation temperature	/
	Additional processing steps (e.g. filtration)	/
	Storage tubes (brand, type, source, catalog number)	/
	Storage temperature	/
	Length of storage (range in years)	/
	Number of freeze-thaw cycles (range)	/
	Thawing temperature (°C)	/
	Thawing duration (minutes)	/
	Presence of hemolysis	/
	Number of samples affected (e.g. <25%, 25-50%)	/
	Method used	/
	Other method used	/
	RBC count (Median, 95% CI, N)	/
	RBC counter brand and type	/
	Spectrophotometry hemoglobin concentration (g/L)	/
	Spectrophotometer brand, model and wavelength measured (e.g. 414 nm)	/
	Hemolized samples were discarded	/
	Presence of platelets	/
	Method used for platelets	/
	Other method used	/

MiBlood-EV		
Plasma/Serum quality control	Platelet marker(s) used (e.g. CD61, CD41)	/
	Concentration (median, 95% CI, N)	/
	Platelet counter instrument brand and type	/
	Flow cytometer brand and type	/
	Flow cytometry size and fluorescence ranges of detection in nanometers and MESF, respectively	/
	Presence of lipoproteins	/
	Method used (WB, ELISA, FC)	/
	Other method used	/
	Spectrophotometry L-index	/
	Spectrophotometer brand, model and wavelength measured (e.g. 700 nm)	/
	WB Marker(s) used (e.g. Apo B)	/
	Western blot images provided in manuscript?	/
	Flow cytometry marker(s) used (e.g. ApoB)	/
	Flow cytometry concentration (median, 95% CI, N)	/
	Flow cytometer brand and type	/
	Flow cytometry size and fluorescence ranges of detection in nanometers and MESF, respectively	/

Other information

General information	
Title	Human platelet lysate-derived exosomes are superior to the lysate at increasing collagen deposition in a rat model of intrinsic aging
Nomenclature used	Exosomes
Application	Therapeutic
EV-track ID	

Collection and storage		
Releasing cell information	Cell type origin	Animalia / / Blood (Plasma/Serum) /
	Passaging	
	Seeding density	cells/cm²
	Cell viability	
	Culture volume	ml
	Culture vessel	
	Oxygen level	%
Culture conditions	Culturing medium	
	Duration of cultivation	
	Harvesting medium	
	Cell count at harvest	cells
Storage and recovery	Condition medium	°C
	EV preparations	N/A °C

EV isolation		
**Category**	**Methods used**	**Details**
Density based	/	/
Size based	/	/
Precipitation based	/	/
Affinity purification	/	/
Chromatography	NA	Size-exclusion chromatography (automatic fraction collector [AFC]izon qEV, Izon Science, New Zealand)
Microfluidics	/	/

EV characterization				
		Parameter	Unit	Method
Quantification	Size & concentration	Particle number	/	/
		Particle size	113 nm	Nano tracking analysis
		Particle/protein ratio	/	/
	Composition	Protein content	/	/
		Lipid content	/	/
		RNA content	/	/
Identity		Category 1- Transmembrane or GPI-anchored proteins associated to plasma membrane and/or endosomes	/	
		Category 2- Cytosolic proteins recovered in EVs	/	
		Category 3- Major components of non-EV co-isolated structures	/	
Visualization		/		

EV function(s)	
Endogenous regeneration	

MiBlood-EV		
Blood study information	Time period of experiment (Years)	/
	Number of samples	/
	Biospecimen type	/
	Biospecimen state	/
	Source of frozen specimen	/
	Patient fasting status	/
	Fasting length (e.g. hours/days)	/
	Anatomical access site	/
	Needle diameter (e.g. gauge)	/
	Blood volume collected (mL)	/
	Plasma anticoagulant	/
	Other plasma anticoagulant	/
	Serum tube type	/
	Serum clotting time (minutes)	/

MiBlood-EV		
Blood collection and processing	Time between collection and first centrifugation (range in hours)	/
	Transport temperature	/
	Transport condition of tubes	/
	Centrifuge brand and model	/
	Bucket rotor type	/
	Number of centrifugation cycles	/
	1st Centrifugation speed (RCF in x g)	/
	1st Rotor brake	/
	1st Centrifugation temperature	/
	2nd Centrifugation speed (RCF in x g)	/
	2nd Rotor brake	/
	2nd Centrifugation temperature	/
	Additional processing steps (e.g. filtration)	/
	Storage tubes (brand, type, source, catalog number)	/
	Storage temperature	/
	Length of storage (range in years)	/
	Number of freeze-thaw cycles (range)	/
	Thawing temperature (°C)	/
	Thawing duration (minutes)	/
	Presence of hemolysis	/
	Number of samples affected (e.g. <25%, 25-50%)	/
	Method used	/
	Other method used	/
	RBC count (Median, 95% CI, N)	/
	RBC counter brand and type	/
	Spectrophotometry hemoglobin concentration (g/L)	/
	Spectrophotometer brand, model and wavelength measured (e.g. 414 nm)	/
	Hemolized samples were discarded	/
	Presence of platelets	/
	Method used for platelets	/
	Other method used	/

MiBlood-EV		
Plasma/Serum quality control	Platelet marker(s) used (e.g. CD61, CD41)	/
	Concentration (median, 95% CI, N)	/
	Platelet counter instrument brand and type	/
	Flow cytometer brand and type	/
	Flow cytometry size and fluorescence ranges of detection in nanometers and MESF, respectively	/
	Presence of lipoproteins	/
	Method used (WB, ELISA, FC)	/
	Other method used	/
	Spectrophotometry L-index	/
	Spectrophotometer brand, model and wavelength measured (e.g. 700 nm)	/
	WB Marker(s) used (e.g. Apo B)	/
	Western blot images provided in manuscript?	/
	Flow cytometry marker(s) used (e.g. ApoB)	/
	Flow cytometry concentration (median, 95% CI, N)	/
	Flow cytometer brand and type	/
	Flow cytometry size and fluorescence ranges of detection in nanometers and MESF, respectively	/

Other information

General information	
Title	Exosomal miRNA-26b-5p from PRP suppresses NETs by targeting MMP-8 to promote diabetic wound healing
Nomenclature used	Exosomes
Application	Therapeutic
EV-track ID	

Collection and storage		
Releasing cell information	Cell type origin	Animalia / / Blood (Plasma/Serum) /
	Passaging	
	Seeding density	cells/cm²
	Cell viability	
	Culture volume	ml
	Culture vessel	
	Oxygen level	%
Culture conditions	Culturing medium	
	Duration of cultivation	
	Harvesting medium	
	Cell count at harvest	cells
Storage and recovery	Condition medium	°C
	EV preparations	N/A °C

EV isolation		
**Category**	**Methods used**	**Details**
Density based	Ultracentrifugation	100,000 g x 70 minutes repeated twice
Size based	/	/
Precipitation based	/	/
Affinity purification	/	/
Chromatography	/	/
Microfluidics	/	/

EV characterization				
		Parameter	Unit	Method
Quantification	Size & concentration	Particle number	7.7x10e9 particles/ml	Nano tracking analysis
		Particle size	30-150 nm	Nano tracking analysis
		Particle/protein ratio	particles/µg	
	Composition	Protein content	/	/
		Lipid content	/	/
		RNA content	/	/
Identity		Category 1- Transmembrane or GPI-anchored proteins associated to plasma membrane and/or endosomes	CD41;CD63	/,
		Category 2- Cytosolic proteins recovered in EVs	TSG101	/,
		Category 3- Major components of non-EV co-isolated structures	/	/,
Visualization		TEM	TEM,	

EV function(s)	
Cytoprotection;Immunomodulation;Endo regeneration	

MiBlood-EV		
Blood study information	Time period of experiment (Years)	/
	Number of samples	/
	Biospecimen type	Plasma
	Biospecimen state	/
	Source of frozen specimen	/
	Patient fasting status	/
	Fasting length (e.g. hours/days)	/
	Anatomical access site	/
	Needle diameter (e.g. gauge)	/
	Blood volume collected (mL)	/
	Plasma anticoagulant	/
	Other plasma anticoagulant	/
	Serum tube type	/
	Serum clotting time (minutes)	/

MiBlood-EV		
Blood collection and processing	Time between collection and first centrifugation (range in hours)	/
	Transport temperature	/
	Transport condition of tubes	/
	Centrifuge brand and model	/
	Bucket rotor type	/
	Number of centrifugation cycles	/
	1st Centrifugation speed (RCF in x g)	/
	1st Rotor brake	/
	1st Centrifugation temperature	/
	2nd Centrifugation speed (RCF in x g)	/
	2nd Rotor brake	/
	2nd Centrifugation temperature	/
	Additional processing steps (e.g. filtration)	/
	Storage tubes (brand, type, source, catalog number)	/
	Storage temperature	/
	Length of storage (range in years)	/
	Number of freeze-thaw cycles (range)	/
	Thawing temperature (°C)	/
	Thawing duration (minutes)	/
	Presence of hemolysis	/
	Number of samples affected (e.g. <25%, 25-50%)	/
	Method used	/
	Other method used	/
	RBC count (Median, 95% CI, N)	/
	RBC counter brand and type	/
	Spectrophotometry hemoglobin concentration (g/L)	/
	Spectrophotometer brand, model and wavelength measured (e.g. 414 nm)	/
	Hemolized samples were discarded	/
	Presence of platelets	/
	Method used for platelets	/
	Other method used	/

MiBlood-EV		
Plasma/Serum quality control	Platelet marker(s) used (e.g. CD61, CD41)	/
	Concentration (median, 95% CI, N)	/
	Platelet counter instrument brand and type	/
	Flow cytometer brand and type	/
	Flow cytometry size and fluorescence ranges of detection in nanometers and MESF, respectively	/
	Presence of lipoproteins	/
	Method used (WB, ELISA, FC)	/
	Other method used	/
	Spectrophotometry L-index	/
	Spectrophotometer brand, model and wavelength measured (e.g. 700 nm)	/
	WB Marker(s) used (e.g. Apo B)	/
	Western blot images provided in manuscript?	/
	Flow cytometry marker(s) used (e.g. ApoB)	/
	Flow cytometry concentration (median, 95% CI, N)	/
	Flow cytometer brand and type	/
	Flow cytometry size and fluorescence ranges of detection in nanometers and MESF, respectively	/

Other information

General information	
Title	Fiber-reinforced gelatin/β-cyclodextrin hydrogels loaded with platelet-rich plasma-derived exosomes for diabetic wound healing
Nomenclature used	Exosomes
Application	Therapeutic
EV-track ID	

Collection and storage		
Releasing cell information	Cell type origin	Animalia / / Blood (Plasma/Serum) /
	Passaging	
	Seeding density	cells/cm²
	Cell viability	
	Culture volume	ml
	Culture vessel	
	Oxygen level	%
Culture conditions	Culturing medium	
	Duration of cultivation	
	Harvesting medium	
	Cell count at harvest	cells
Storage and recovery	Condition medium	°C
	EV preparations	N/A °C

EV isolation		
**Category**	**Methods used**	**Details**
Density based	Ultracentrifugation	10000 g x 30 minutes, 110,000 g x 70 minutes repeated twice
Size based	/	/
Precipitation based	/	/
Affinity purification	/	/
Chromatography	/	/
Microfluidics	/	/

EV characterization				
		Parameter	Unit	Method
Quantification	Size & concentration	Particle number	/	/
		Particle size	30-150 nm	Nano tracking analysis
		Particle/protein ratio	/	/
	Composition	Protein content	/	/
		Lipid content	/	/
		RNA content	/	/
Identity		Category 1- Transmembrane or GPI-anchored proteins associated to plasma membrane and/or endosomes	CD81	/,
		Category 2- Cytosolic proteins recovered in EVs	HSC70;TSG101	/,
		Category 3- Major components of non-EV co-isolated structures	/	/,
Visualization		TEM	TEM,	

EV function(s)	
Cytoprotection;Vasculogenesis and Angiogenesis;Endogenous regeneration	

MiBlood-EV		
Blood study information	Time period of experiment (Years)	/
	Number of samples	/
	Biospecimen type	Plasma
	Biospecimen state	Fresh
	Source of frozen specimen	/
	Patient fasting status	/
	Fasting length (e.g. hours/days)	/
	Anatomical access site	/
	Needle diameter (e.g. gauge)	/
	Blood volume collected (mL)	/
	Plasma anticoagulant	/
	Other plasma anticoagulant	ACD-A
	Serum tube type	/

MiBlood-EV		
Blood collection and processing	Serum clotting time (minutes)	/
	Time between collection and first centrifugation (range in hours)	/
	Transport temperature	/
	Transport condition of tubes	/
	Centrifuge brand and model	/
	Bucket rotor type	/
	Number of centrifugation cycles	/
	1st Centrifugation speed (RCF in x g)	160
	1st Rotor brake	/
	1st Centrifugation temperature	/
	2nd Centrifugation speed (RCF in x g)	250
	2nd Rotor brake	/
	2nd Centrifugation temperature	/
	Additional processing steps (e.g. filtration)	/
	Storage tubes (brand, type, source, catalog number)	/
	Storage temperature	/
	Length of storage (range in years)	/
	Number of freeze-thaw cycles (range)	/
	Thawing temperature (°C)	/
	Thawing duration (minutes)	/
	Presence of hemolysis	No
	Number of samples affected (e.g. <25%, 25-50%)	/
	Method used	/
	Other method used	/
	RBC count (Median, 95% CI, N)	/
	RBC counter brand and type	/
	Spectrophotometry hemoglobin concentration (g/L)	/
	Spectrophotometer brand, model and wavelength measured (e.g. 414 nm)	/
	Hemolized samples were discarded	/
	Presence of platelets	/
	Method used for platelets	/

MiBlood-EV		
Plasma/Serum quality control	Other method used	/
	Platelet marker(s) used (e.g. CD61, CD41)	/
	Concentration (median, 95% CI, N)	/
	Platelet counter instrument brand and type	/
	Flow cytometer brand and type	/
	Flow cytometry size and fluorescence ranges of detection in nanometers and MESF, respectively	/
	Presence of lipoproteins	/
	Method used (WB, ELISA, FC)	/
	Other method used	/
	Spectrophotometry L-index	/
	Spectrophotometer brand, model and wavelength measured (e.g. 700 nm)	/
	WB Marker(s) used (e.g. Apo B)	/
	Western blot images provided in manuscript?	/
	Flow cytometry marker(s) used (e.g. ApoB)	/
	Flow cytometry concentration (median, 95% CI, N)	/
	Flow cytometer brand and type	/
	Flow cytometry size and fluorescence ranges of detection in nanometers and MESF, respectively	/

Other information

General information	
Title	Wound healing effects of a Curcuma zedoaria polysaccharide with platelet-rich plasma exosomes assembled on chitosan/silk hydrogel sponge in a diabetic rat model
Nomenclature used	Exosomes
Application	Therapeutic
EV-track ID	

Collection and storage		
Releasing cell information	Cell type origin	Animalia / / Blood (Plasma/Serum) /
	Passaging	
	Seeding density	cells/cm²
	Cell viability	
	Culture volume	ml
	Culture vessel	
	Oxygen level	%
Culture conditions	Culturing medium	
	Duration of cultivation	
	Harvesting medium	
	Cell count at harvest	cells
Storage and recovery	Condition medium	°C
	EV preparations	N/A °C

EV isolation		
**Category**	**Methods used**	**Details**
Density based	/	/
Size based	/	/
Precipitation based	/	/
Affinity purification	/	/
Chromatography	/	/
Microfluidics	/	/

EV characterization				
		Parameter	Unit	Method
Quantification	Size & concentration	Particle number	/	/
		Particle size	/	/
		Particle/protein ratio	/	/
	Composition	Protein content	/	/
		Lipid content	/	/
		RNA content	/	/
Identity		Category 1- Transmembrane or GPI-anchored proteins associated to plasma membrane and/or endosomes	/	
		Category 2- Cytosolic proteins recovered in EVs	/	
		Category 3- Major components of non-EV co-isolated structures	/	
Visualization		/		

EV function(s)	
Vasculogenesis and Angiogenesis;Endogenous regeneration	

MiBlood-EV		
Blood study information	Time period of experiment (Years)	/
	Number of samples	/
	Biospecimen type	Plasma
	Biospecimen state	/
	Source of frozen specimen	/
	Patient fasting status	/
	Fasting length (e.g. hours/days)	/
	Anatomical access site	/
	Needle diameter (e.g. gauge)	/
	Blood volume collected (mL)	/
	Plasma anticoagulant	/
	Other plasma anticoagulant	/
	Serum tube type	/

MiBlood-EV		
Blood collection and processing	Serum clotting time (minutes)	/
	Time between collection and first centrifugation (range in hours)	/
	Transport temperature	/
	Transport condition of tubes	/
	Centrifuge brand and model	/
	Bucket rotor type	/
	Number of centrifugation cycles	/
	1st Centrifugation speed (RCF in x g)	/
	1st Rotor brake	/
	1st Centrifugation temperature	/
	2nd Centrifugation speed (RCF in x g)	/
	2nd Rotor brake	/
	2nd Centrifugation temperature	/
	Additional processing steps (e.g. filtration)	/
	Storage tubes (brand, type, source, catalog number)	/
	Storage temperature	/
	Length of storage (range in years)	/
	Number of freeze-thaw cycles (range)	/
	Thawing temperature (°C)	/
	Thawing duration (minutes)	/
	Presence of hemolysis	/
	Number of samples affected (e.g. <25%, 25-50%)	/
	Method used	/
	Other method used	/
	RBC count (Median, 95% CI, N)	/
	RBC counter brand and type	/
	Spectrophotometry hemoglobin concentration (g/L)	/
	Spectrophotometer brand, model and wavelength measured (e.g. 414 nm)	/
	Hemolized samples were discarded	/
	Presence of platelets	/
	Method used for platelets	/

MiBlood-EV		
Plasma/Serum quality control	Other method used	/
	Platelet marker(s) used (e.g. CD61, CD41)	/
	Concentration (median, 95% CI, N)	/
	Platelet counter instrument brand and type	/
	Flow cytometer brand and type	/
	Flow cytometry size and fluorescence ranges of detection in nanometers and MESF, respectively	/
	Presence of lipoproteins	/
	Method used (WB, ELISA, FC)	/
	Other method used	/
	Spectrophotometry L-index	/
	Spectrophotometer brand, model and wavelength measured (e.g. 700 nm)	/
	WB Marker(s) used (e.g. Apo B)	/
	Western blot images provided in manuscript?	/
	Flow cytometry marker(s) used (e.g. ApoB)	/
	Flow cytometry concentration (median, 95% CI, N)	/
	Flow cytometer brand and type	/
	Flow cytometry size and fluorescence ranges of detection in nanometers and MESF, respectively	/

Other information

General information	
Title	Ultrasound-Targeted Microbubble Destruction Assisted Delivery of Platelet-Rich Plasma-Derived Exosomes Promoting Peripheral Nerve Regeneration
Nomenclature used	Exosomes
Application	Therapeutic
EV-track ID	

Collection and storage		
Releasing cell information	Cell type origin	Animalia / / Blood (Plasma/Serum) /
	Passaging	
	Seeding density	cells/cm²
	Cell viability	
	Culture volume	ml
	Culture vessel	
	Oxygen level	%
Culture conditions	Culturing medium	
	Duration of cultivation	
	Harvesting medium	
	Cell count at harvest	cells
Storage and recovery	Condition medium	°C
	EV preparations	-80 °C

EV isolation		
**Category**	**Methods used**	**Details**
Density based	Ultracentrifugation	100,000 g x 70 minutes
Size based	Ultrafiltration	0.22 μm filter
Precipitation based	ExoQuick	ExoQuick Plasma Prep Exosome Precipitation Kit
Affinity purification	/	/
Chromatography	/	/
Microfluidics	/	/

EV characterization				
		Parameter	Unit	Method
Quantification	Size & concentration	Particle number	5.95x10e10 particles/ml	FCM (e.g. NanoFCM)
		Particle size	50-100 nm	FCM (e.g. NanoFCM)
		Particle/protein ratio	particles/µg	
	Composition	Protein content	/	/
		Lipid content	/	/
		RNA content	/	/
Identity		Category 1- Transmembrane or GPI-anchored proteins associated to plasma membrane and/or endosomes	CD41;CD63;CD81;CD9	/,
		Category 2- Cytosolic proteins recovered in EVs	/	/,
		Category 3- Major components of non-EV co-isolated structures	/	/,
Visualization		TEM	TEM,	

EV function(s)	
Endogenous regeneration	

MiBlood-EV		
Blood study information	Time period of experiment (Years)	/
	Number of samples	/
	Biospecimen type	Plasma
	Biospecimen state	Fresh
	Source of frozen specimen	/
	Patient fasting status	/
	Fasting length (e.g. hours/days)	/
	Anatomical access site	/
	Needle diameter (e.g. gauge)	/
	Blood volume collected (mL)	/
	Plasma anticoagulant	/
	Other plasma anticoagulant	ACD-A
	Serum tube type	/
	Serum clotting time (minutes)	/

MiBlood-EV		
Blood collection and processing	Time between collection and first centrifugation (range in hours)	/
	Transport temperature	/
	Transport condition of tubes	/
	Centrifuge brand and model	/
	Bucket rotor type	/
	Number of centrifugation cycles	/
	1st Centrifugation speed (RCF in x g)	160
	1st Rotor brake	/
	1st Centrifugation temperature	/
	2nd Centrifugation speed (RCF in x g)	250
	2nd Rotor brake	/
	2nd Centrifugation temperature	/
	Additional processing steps (e.g. filtration)	/
	Storage tubes (brand, type, source, catalog number)	/
	Storage temperature	/
	Length of storage (range in years)	/
	Number of freeze-thaw cycles (range)	/
	Thawing temperature (°C)	/
	Thawing duration (minutes)	/
	Presence of hemolysis	/
	Number of samples affected (e.g. <25%, 25-50%)	/
	Method used	/
	Other method used	/
	RBC count (Median, 95% CI, N)	/
	RBC counter brand and type	/
	Spectrophotometry hemoglobin concentration (g/L)	/
	Spectrophotometer brand, model and wavelength measured (e.g. 414 nm)	/
	Hemolized samples were discarded	/
	Presence of platelets	/
	Method used for platelets	/
	Other method used	/

MiBlood-EV		
Plasma/Serum quality control	Platelet marker(s) used (e.g. CD61, CD41)	/
	Concentration (median, 95% CI, N)	/
	Platelet counter instrument brand and type	/
	Flow cytometer brand and type	/
	Flow cytometry size and fluorescence ranges of detection in nanometers and MESF, respectively	/
	Presence of lipoproteins	/
	Method used (WB, ELISA, FC)	/
	Other method used	/
	Spectrophotometry L-index	/
	Spectrophotometer brand, model and wavelength measured (e.g. 700 nm)	/
	WB Marker(s) used (e.g. Apo B)	/
	Western blot images provided in manuscript?	/
	Flow cytometry marker(s) used (e.g. ApoB)	/
	Flow cytometry concentration (median, 95% CI, N)	/
	Flow cytometer brand and type	/
	Flow cytometry size and fluorescence ranges of detection in nanometers and MESF, respectively	/

Other information
