# Supplementary material for: Therapeutic Potential of Extracellular Vesicles (Exosomes) Derived From Platelet‐Rich Plasma: A Literature Review
Source: J Cosmet Dermatol. 2024 Dec 1;24(2):e16709. doi: 10.1111/jocd.16709 (PMC11845942; doi:10.1111/jocd.16709)
Supplement: Supplementary file 2 — Data S2. [file JOCD-24-e16709-s001.docx]

**Table S1.** Platelets isolation and exosome characteristics from included pre-clinical studies.

| **Study** | **Platelets (/mL)** | **Exosome Isolation** | **Exosomes**  **(/mL)** | **Diameter (nm), mean (range)** | **Markers** |
| --- | --- | --- | --- | --- | --- |
| Chen 2023 [1]^1^ | 1.1 x 10^9^ | Ultracentrifugation  Filtration | - | 88.01  (50-150; SD: 21.01) | (+):CD41, CD63, Flotillin, TSG101  (-): Calnexin |
| Rui 2024^2^ | 1.6 x 10^9^ | Ultracentrifugation | 7.7 x 10^9^ | 80.42  (30-150; SD: 16) | (+): CD63, TSG101, CD41  (-): Calnexin |
| Bakadia 2023^3^ | 3.8 x 10^9^ | Ultracentrifugation  Filtration | 6.9 x 10^10^ | 60 (SD: 10) | (+): CD9, CD63, CD81 |
| Cao 2023^4^ | - | Ultracentrifugation  Filtration | - | 117.5 | (+): CD9, CD61, CD81, CD41 |
| Chen 2023 [2]^5^ | - | Ultracentrifugation  Precipitation  Filtration | - | 40-100 | - |
| Lovisolo 2020^6^ | 4.0 x 10^6^ | Ultracentrifugation | - | - | - |
| Gnomes 2022^7^ | - | Filtration  TFF  SEC | 2.2 x 10^12^ | - | (+): CD9, CD63 |
| Guo 2017^8^ | - | Ultracentrifugation  Filtration | - | 66.8  (40-100; SD: 31.61) | (+):CD9, CD63, CD81, CD41 |
| Shu 2023^9^ | - | Ultracentrifugation | - | 30-150 | (+): CD81, HSP70, TSG101 |
| Yi 2023^10^ | - | Precipitation  Filtration  Ultracentrifugation | 6.0 x 10^10^ | 83.76  (50-100) | (+): CD9, CD63, CD81, CD41 |
| Xu 2018^11^ | - | - | - | - | - |
| Nilforoushzadeh 2020^12^ | 1.0 x 10^9^ | Ultracentrifugation | - | 147  (30-170) | (+): CD9, CD63, CD81, TSG101  (-): Calnexin |
| Nilforoushzadeh 2021^13^ | 1.0 x 10^9^ | Ultracentrifugation | - | 147  (30-150) | (+): CD9, CD63, CD81, TSG101  (-): Calnexin |
| Rosmarwati 2023^14^ | - | SEC | 1.4 x 10^9^ | 113 | - |
| SEC, size-exclusion chromatography; SD, standard deviation; TFF, tangential-flow filtration  (+) denotes positive surface markers; (-) denotes negative surface markers | | | | | |

**References**

1. Chen T, Song P, He M, et al. Sphingosine-1-phosphate derived from PRP-Exos promotes angiogenesis in diabetic wound healing via the S1PR1/AKT/FN1 signalling pathway. *Burn Trauma*. 2023;11. doi:10.1093/burnst/tkad003

2. Rui S, Dai L, Zhang X, et al. Exosomal miRNA-26b-5p from PRP suppresses NETs by targeting MMP-8 to promote diabetic wound healing. *J Control Release*. 2024;372(June):221-233. doi:10.1016/j.jconrel.2024.06.050

3. Bakadia BM, Qaed Ahmed AA, Lamboni L, et al. Engineering homologous platelet-rich plasma, platelet-rich plasma-derived exosomes, and mesenchymal stem cell-derived exosomes-based dual-crosslinked hydrogels as bioactive diabetic wound dressings. *Bioact Mater*. 2023;28(December 2022):74-94. doi:10.1016/j.bioactmat.2023.05.002

4. Cao W, Meng X, Cao F, Wang J, Yang M. Exosomes derived from platelet-rich plasma promote diabetic wound healing via the JAK2/STAT3 pathway. *iScience*. 2023;26(11). doi:10.1016/j.isci.2023.108236

5. Chen C, Wang Q, Li D, Qi Z, Chen Y, Wang S. MALAT1 participates in the role of platelet-rich plasma exosomes in promoting wound healing of diabetic foot ulcer. *Int J Biol Macromol*. 2023;238(November 2022). doi:10.1016/j.ijbiomac.2023.124170

6. Lovisolo F, Carton F, Gino S, Migliario M, Renò F. Platelet rich plasma-derived microvesicles increased in vitro wound healing. *Eur Rev Med Pharmacol Sci*. 2020;24(18):9658-9664. doi:10.26355/eurrev_202009_23055

7. Gomes FG, Andrade AC, Wolf M, et al. Synergy of Human Platelet-Derived Extracellular Vesicles with Secretome Proteins Promotes Regenerative Functions. *Biomedicines*. 2022;10(2). doi:10.3390/biomedicines10020238

8. Guo SC, Tao SC, Yin WJ, Qi X, Yuan T, Zhang CQ. Exosomes derived from platelet-rich plasma promote the re-epithelization of chronic cutaneous wounds via activation of YAP in a diabetic rat model. *Theranostics*. 2017;7(1):81-96. doi:10.7150/thno.16803

9. Shu QH, Zuo RT, Chu M, et al. Fiber-reinforced gelatin/β-cyclodextrin hydrogels loaded with platelet-rich plasma-derived exosomes for diabetic wound healing. *Biomater Adv*. 2023;154(September):213640. doi:10.1016/j.bioadv.2023.213640

10. Yi D, Zhang Y, Li M, et al. Ultrasound-Targeted Microbubble Destruction Assisted Delivery of Platelet-Rich Plasma-Derived Exosomes Promoting Peripheral Nerve Regeneration. *Tissue Eng - Part A*. 2023;29(23-24):645-662. doi:10.1089/ten.tea.2023.0133

11. Xu N, Wang L, Guan J, et al. Wound healing effects of a Curcuma zedoaria polysaccharide with platelet-rich plasma exosomes assembled on chitosan/silk hydrogel sponge in a diabetic rat model. *Int J Biol Macromol*. 2018;117:102-107. doi:10.1016/j.ijbiomac.2018.05.066

12. Nilforoushzadeh MA, Aghdami N, Taghiabadi E. Human Hair Outer Root Sheath Cells and Platelet-Lysis Exosomes Promote Hair Inductivity of Dermal Papilla Cell. *Tissue Eng Regen Med*. 2020;17(4):525-536. doi:10.1007/s13770-020-00266-4

13. Nilforoushzadeh MA, Aghdami N, Taghiabadi E. Effects of adipose-derived stem cells and platelet-rich plasma exosomes on the inductivity of hair dermal papilla cells. *Cell J*. 2021;23(5):576-583. doi:10.22074/cellj.2021.7352

14. Rosmarwati E, Ellistasari EY, Kusumawardani A, et al. Human platelet lysate-derived exosomes are superior to the lysate at increasing collagen deposition in a rat model of intrinsic aging. *J Appl Pharm Sci*. 2023;13(6):211-216. doi:10.7324/JAPS.2023.125707
